# Construction of an HLA Classifier for Early Diagnosis, Prognosis, and Recognition of Immunosuppression in Sepsis by Multiple Transcriptome Datasets

**DOI:** 10.3389/fphys.2022.870657

**Published:** 2022-05-24

**Authors:** Zhen Chen, Rui Chen, Yangpeng Ou, Jianhai Lu, Qianhua Jiang, Genglong Liu, Liping Wang, Yayun Liu, Zhujiang Zhou, Ben Yang, Liuer Zuo

**Affiliations:** ^1^ Department of Intensive Care Unit, Shunde Hospital, Southern Medical University (The First People’s Hospital of Shunde), Foshan, China; ^2^ Department of Medical Intensive Care Unit, General Hospital of Southern Theater Command, Guangzhou, China; ^3^ Department of Oncology, Huizhou Third People’s Hospital, Guangzhou Medical University, Huizhou, China; ^4^ Department of Pathology, The Third Affiliated Hospital of Guangdong Medical University (Longjiang Hospital of Shunde District), Foshan, China; ^5^ Department of Otorhinolaryngology Head and Neck Surgery, The First Affiliated Hospital of Hainan Medical University, Haikou, China; ^6^ Department of Endocrinology, GuiYang Huaxi District People’s Hospital, Guiyang, China; ^7^ Department of Burn Surgery, Huizhou Municipal Central Hospital, Huizhou, China

**Keywords:** sepsis, HLA genes, immune infiltration, immunosuppression, model

## Abstract

**Background:** Sepsis is a clinical syndrome, due to a dysregulated inflammatory response to infection. Accumulating evidence shows that human leukocyte antigen (HLA) genes play a key role in the immune responses to sepsis. Nevertheless, the effects of HLA genes in sepsis have still not been comprehensively understood.

**Methods:** A systematical search was performed in the Gene Expression Omnibus (GEO) and ArrayExpress databases from inception to 10 September 2021. Random forest (RF) and modified Lasso penalized regression were conducted to identify hub genes in multi-transcriptome data, thus we constructed a prediction model, namely the HLA classifier. ArrayExpress databases, as external validation, were utilized to evaluate its diagnostic, prognostic, and predictive performance. Immune cell infiltration score was calculated via CIBERSORTx tools and single-sample gene set enrichment analysis (ssGSEA). Gene set variation analysis (GSVA) and ssGSEA were conducted to determine the pathways that are significantly enriched in different subgroups. Next, we systematically correlated the HLA classifier with immunological characteristics from multiple perspectives, such as immune-related cell infiltration, pivotal molecular pathways, and cytokine expression. Finally, quantitative real-time polymerase chain reaction (qRT-PCR) was conducted to validate the expression level of HLA genes in clinical samples.

**Results:** A total of nine datasets comprising 1,251 patients were included. Based on RF and modified Lasso penalized regression in multi-transcriptome datasets, five HLA genes (B2M, HLA-DQA1, HLA-DPA1, TAP1, and TAP2) were identified as hub genes, which were used to construct an HLA classifier. In the discovery cohort, the HLA classifier exhibited superior diagnostic value (AUC = 0.997) and performed better in predicting mortality (AUC = 0.716) than clinical characteristics or endotypes. Encouragingly, similar results were observed in the ArrayExpress databases. In the E-MTAB-7581 dataset, the use of hydrocortisone in the HLA high-risk subgroup (OR: 2.84, 95% CI 1.07–7.57, *p* = 0.037) was associated with increased risk of mortality, but not in the HLA low-risk subgroup. Additionally, immune infiltration analysis by CIBERSORTx and ssGSEA revealed that B cells, activated dendritic cells, NK cells, T helper cells, and infiltrating lymphocytes (ILs) were significantly richer in HLA low-risk phenotypes, while Tregs and myeloid-derived suppressor cells (MDSCs) were more abundant in HLA high-risk phenotypes. The HLA classifier was significantly negatively correlated with B cells, activated dendritic cells, NK cells, T helper cells, and ILs, yet was significantly positively correlated with Tregs and MDSCs. Subsequently, molecular pathways analysis uncovered that cytokine-cytokine receptor (CCR) interaction, human leukocyte antigen (HLA), and antigen-presenting cell (APC) co-stimulation were significantly enriched in HLA low-risk endotypes, which was significantly negatively correlated with the HLA classifier in multi-transcriptome data. Finally, the expression levels of several cytokines (IL-10, IFNG, TNF) were significantly different between the HLA subgroups, and the ratio of IL-10/TNF was significantly positively correlated with HLA score in multi-transcriptome data. Results of qRT-PCR validated the higher expression level of B2M as well as lower expression level of HLA-DQA1, HLA-DPA1, TAP1, and TAP2 in sepsis samples compared to control sample.

**Conclusion:** Based on five HLA genes, a diagnostic and prognostic model, namely the HLA classifier, was established, which is closely correlated with responses to hydrocortisone and immunosuppression status and might facilitate personalized counseling for specific therapy.

## Key Messages

1) To the best of our knowledge, this is the first comprehensive study to explore the HLA family based on multiple transcriptome expression profiles in all-cause sepsis, leading to the discovery of novel biomarkers to develop a diagnostic and prognostic model, thus elucidating the model and immune system (immune cells infiltration, immune-related pathways, and cytokines) to find its additional clinical implications.
2) Based on random forest and modified Lasso penalized regression, a diagnostic and prognostic model (HLA classifier) was constructed, which could be a robust tool to diagnose sepsis earlier and to identify patients at risk of a poor or even fatal outcome. Additionally, the HLA classifier is closely correlated with responses to hydrocortisone and may be useful for clinicians to tailor treatment decisions. According to immune cell infiltration, immune-related pathways, and cytokines level, the HLA classifier could efficiently reflect immunological status, which may help guide immune-modulating agents to achieve immune homeostasis.
3) Results of qRT-PCR validated a higher expression level of B2M as well as a lower expression level of HLA-DQA1, HLA-DPA1, TAP1, and TAP2 in sepsis samples compared to control samples, which were in accordance with the results of bioinformatics analyses derived from the GEO datasets.

## Introduction

Sepsis, a life-threatening syndrome characterized by organ failure after infection, is caused by a dysregulated host response to infection (Singer et al., 2016). Clinical epidemiological analyses show that the estimated national cases and in-hospital mortality cases of sepsis were approximately 48.9 million and 11.0 million, respectively, representing one-fifth of all causes of death, and making it one of the major socioeconomic burdens all over the world (Rudd et al., 2020). In the past decade, according to the recommendations of the Surviving Sepsis Campaign (SSC) for the management of sepsis patients, the mortality rate decreased from about 37%–25%, whereas this figure is still too high to be acceptable (Fleischmann et al., 2016). To fight the global burden of sepsis, given the lack of obvious and nonspecific clinical signs in early-stage disease, early diagnosis and appropriate treatment is critical to improve patients’ outcomes, on account of the fact that each hour of delay in initiating treatment is associated with increased mortality rates (Ferrer et al., 2014). Importantly, classification and identification of a patient at high risk may aid clinicians to screen and identify individuals who are most likely to benefit from additional monitoring and treatment, or to detect an immunosuppressed state which could benefit from targeted immunostimulating therapies, and eventually improve patient prognosis.

As sepsis is a highly intricate condition and its clinical evaluation is often challenging, the additional usage of biomarkers for rapid diagnosis that help pinpoint high-risk patients is an attractive solution. Currently, several biomarkers, such as C-reactive protein (CRP), which is characterized as a inflammatory marker, and procalcitonin (PCT), which serves as a marker of bacteremia, have been widely utilized as acute phase reactants in critically ill patients, yet their diagnostic and prognostic performance for sepsis are suboptimal (Henriquez-Camacho and Losa, 2014). Recently, the quick sequential organ failure (qSOFA) score was introduced as a bedside standard based on three clinical elements, which was generated through a data-driven approach. However, it has been controversial since it was proposed, in terms of the diagnosis, it has high sensitivity and low specificity, yet with regard to the prognosis, it has low sensitivity and high specificity, which makes its implementation problematic (Simpson, 2017). To date, none of the signatures of the immune response or circulating blood biomarkers that have been investigated detect sepsis quickly enough or recognize high-risk patients with an acceptable certainty, which was ascribed to the heterogeneity and complex pathophysiology of sepsis. To a certain extent, the heterogeneity can be related to the differential expression of thousands of genes in response to infectious stimuli (Zhai et al., 2020). Hence, transcriptomics, as promising new biomarkers, can provide important predictive and prognostic information.

Pathophysiologically, human leukocyte antigen (HLA), cross-link innate and adaptive, plays an important role in recognition, processing, and presentation of protein antigens (such as organisms) to cognate T cells, NK cells, etc., therefore, initiating an immune response, which is involved in the pathogenesis of sepsis (Koşaloğlu-Yalçın et al., 2018). HLA-DR, which belongs to HLA class II and is located on chromosome 6, plays a crucial role in modulating immune responses. Diminished monocyte HLA-DR (mHLA-DR) expression on the cell surface was associated with increased risk of death of septic patients or adverse outcomes and increased susceptibility to nosocomial/secondary infections (Hotchkiss et al., 2016). It is noteworthy that mHLA-DR is considered an effective indicator of the general immunoparalysis state of patients. However, on account of inferior specificity and sensitivity, the capacity of HLA-DR expression monitoring to predict mortality is not completely recognized (Monneret et al., 2006). Currently, no single biomarker can be useful for diagnosing sepsis, prognosis, and disease monitoring due to patient heterogeneity. A panel of markers (“sepsis signature”) seems to be able to offer better predictive and prognostic purposes. The availability of genome-sequencing data opened up the possibility of comprehensively investigating all HLA gene alterations of sepsis, resulting in the discovery of new biomarkers for early identification, disease surveillance, and to guide specific adjuvant therapy.

A comprehensive characterization of the HLA family in the adult host response to all-cause sepsis has not previously been done. In our current study, based on random forest (RF) and modified Lasso penalized regression, we identified hub HLA genes, and constructed a prediction model, namely the HLA classifier. Subsequently, the predictive and prognostic values of the model were tested in independent validation cohorts from ArrayExpress databases. Finally, we systematically correlated the HLA classifier with immunological characteristics from multiple perspectives, such as immune-related cell infiltration, pivotal molecular pathways, and cytokine expression.

## Materials and Methods

### Sample Selection, Data Acquisition, and Processing

A comprehensive search was performed in Gene Expression Omnibus (GEO) (http://www.ncbi.nlm.nih.gov/geo) and ArrayExpress (http://www.ncbi.nlm.nih.gov/geo) databases from 9 July 2005 to 10 September 2021 to identify relevant transcriptomic profiling datasets. The inclusion criteria were the following: expression profiling by array or high-throughput sequencing: sepsis; organism: homo sapiens; samples size more than 50; adult patients (more than 18 years old). Ultimately, six GEO datasets, as discovery cohorts, and three ArrayExpress databases, as external validation cohorts, fulfilled our eligibility criteria and were included for both qualitative and quantitative analysis. The basic information of these microarray datasets is listed in Table 1. Additionally, a panel of 28 HLA genes was collected from published studies (Schaafsma et al., 2021) (Supplementary Table S1). All data were normalized with the edgeR package or Limma package in the R computing environment.

**TABLE 1:** Dataset included in the study.

Accession	Cohort description	Timing of gene expression proﬁling	Country	Normal sample	Mortality/sepsis sample	Sample type
GSE65682	Sepsis due to CAP and HAP + AS	On ICU admission	Netherlands and United Kingdom	42	48/231	Whole blood
GSE54514	Sepsis	Within 24 h of ICU admission	Australia	18	9/35	Whole blood
GSE57065	Septic shock	On ICU admission	France	25	-/28	Whole blood
GSE63042	Sepsis	Day of enrollment upon presentation to the ED	United States	—	28/106	Whole blood
GSE69528	Sepsis due to CAP	On ICU admission	United States	55	-/83	Whole blood
GSE95233	Septic shock	Day 1 of ICU admission	France	22	34/51	Whole blood
E-MTAB-4421	Septic shock	On ICU admission	United Kingdom	—	56/265	Whole blood
E-MTAB-4451	Sepsis due to CAP	On ICU admission	United Kingdom	—	57/114	Whole blood
E-MTAB-7581	Septic shock	At enrollment	United Kingdom	—	48/176	Whole blood

Abbreviations: CAP, community acquired pneumonia; HAP, hospital acquired pneumonia; AS, abdominal sepsis; ICU, intensive care medicine; ED, emergency room.

### Clinical Specimens

Peripheral blood mononuclear cells (PBMCs) were collected from 50 clinical samples, including 25 sepsis samples and 25 healthy controls. The study was reviewed and approved by the institutional review board (Ethics Committee) of the Shunde Hospital, Southern Medical University (the First People’s Hospital of Shunde).

### Identification of Hub HLA Genes and Construction of an HLA Classifier

To select out convincing hub HLA genes, machine learning approaches, including modified Lasso penalized regression and RF (random forest), were adopted. A Lasso regression was performed with 10-fold cross-validation to identify candidate HLA genes and was run for 1,000 cycles to select feature variables based on minimum criteria or 1—s.e. criteria. RF (random forest), a tree-based ensemble comprised of tree-structured classifiers, was established to select feature variables via the package “randomForest” with minimum error regression trees. The importance of variables was ranked using IncNodePurity. The real hub HLA genes were obtained from the intersection of the result of Lasso and RF (GSE65682, GSE63042, and GSE95233 datasets), which was used to develop a prediction model, namely the HLA classifier. The HLA score was generated through a linear combination of coefficients from logistic regression and the relative expression of each HLA. According to this formula, each patient’s HLA score was calculated, and patients were classified into low-risk or high-risk groups on the basis of the optimal cut-off value with the maximal sensitivity and specificity in a receiver operating characteristic (ROC) curve.

### Diagnostic and Prognostic Value of the HLA Classifier

ROC analysis using the pROC package was carried out to evaluate diagnostic performance with sepsis as the endpoint. Regarding a prognostic aspect, first univariate and multivariate logistic regression analyses were utilized to adjudicate whether the predictive ability of the HLA classifier remained independent of other clinical features [including age, sex, diabetes mellitus (DM), sepsis response signature (SRS), the Molecular Diagnosis and Risk Stratification of Sepsis (MARS), and Acute Physiology and Chronic Health Evaluation (APACHE II)] in multiple datasets. Then the prognostic value of the HLA classifier was compared against age, SRS, MARS, and APACHE II in the discovery and external validation cohorts.

### Clinical Usefulness of the HLA Classifier

We explored whether there was an interaction between the HLA classifier and the treatment (vasopressin versus norepinephrine; hydrocortisone versus placebo) in logistic regression models, by using the binary mortality outcome as the response variable in the E-MTAB-7581 dataset. Additionally, to evaluate the clinical value of the HLA classifier, decision curve analysis (DCA), calculating the net benefit for a range of threshold probabilities which places benefits and harms on the same scale (Vickers and Elkin, 2006), was utilized to compare age, SRS, MARS, and APACHE II in the discovery and external validation cohorts.

### Evaluation of Immune Cell Infiltration by CIBERSORTx and ssGSEA

To evaluate relative abundance of immune infiltrates, CIBERSORTx (https://cibersort.stanford.edu/) (Newman et al., 2015), which transforms the normalized gene expression matrix into the composition of infiltrating immune cells, and is a kind of deconvolution algorithm that utilizes 1,000 iterations, was used. We filtered out samples with a CIBERSORTx output of a *p-*value more than 0.05 for accurate forecast of immune cell composition. The ggplot2 package was used to generate bar graphs visualizing the content of 22 types of infiltrating immune cells in each sample and violin plots were used to display variance analysis of immune cells between HLA subgroups.

The GSVA package in R was used to conduct ssGSEA on a metagene set of 26 immune cell subtypes (Supplementary Table S2) that are representative of specific immune cells (Bindea et al., 2013). To determine differential immune cell subtypes between the two subgroups (*p*-value < 0.05), the two-tailed Wilcoxon test was utilized to analyze the immunoscores, and violin plots were used to visualize the results. Additionally, we explored the correlation between the HLA classifier and immune cells by Spearman correlation analyses in multiple transcriptome datasets. A *p <* 0.05 would be considered statistically significant.

### Immune and Molecular Function Between the HLA Subgroups by GSVA and ssGSEA

GSVA, which converts genes from a sample matrix into predefined gene sets without a priori knowledge of experiment design, is a non-parametric unsupervised approach. The KEGG gene sets (c2.cp.kegg.v7.4.symbols.gmt), which were downloaded from the Molecular Signatures Database (MSigDB) (http://software.broadinstitute.org/gsea/index.jsp) (Hänzelmann et al., 2013), were used to estimate variation of pathway activity in each sample. The significantly enriched pathways in KEGG gene sets were set at *p*-value < 0.05 and enrichment score change >1.0. Additionally, ssGSEA, which generates an enrichment score to signify the levels of absolute enrichment of a metagene set within certain gene signatures in each sample, was applied to evaluate the enrichment degree of immune-related pathways (Bindea et al., 2013) in current immunology research. Supplementary Table S3 lists the metagene set. Additionally, we explored the relationship between the HLA classifier and pivotal molecular pathways by Spearman correlation analyses in multiple transcriptome datasets. A *p<*0.05 would be considered statistically significant.

### Analyses of the Cytokines

A panel of 27 clinically detectable inflammatory cytokines was collected from published studies (Dong et al., 2019). To further define cytokine expression between the HLA subgroups, two-tailed variance analysis was conducted. Additionally, we explored the relationship between the HLA classifier and cytokine expression level by Spearman correlation analyses in multiple transcriptome datasets. A *p<*0.05 would be considered statistically significant.

### Quantitative Real-Time PCR (qRT-PCR)

Following the manufacturer’s protocol, Trizol (Invitrogen) was used to extract total RNA from peripheral blood mononuclear cells (PBMCs). Reverse transcription of RNA was completed using a RevertAid RT Reverse Transcription Kit (Thermo Scientific). Quantitative PCR was performed using a PowerUp^™^ SYBR^™^ Green Master Mix (Thermo Scientific). The results were standardized with GAPDH. Quantitative reverse transcription PCR was conducted using the ABI 7500 real-time PCR system (Applied Biosystems, Foster City, CA, United States). Fold change was determined as 2^−△△Ct^ in gene expression. Gene-specific PCR primers are listed in Supplementary Table S4.

### Statistical Analysis

R software (R version 3.6.1) was utilized to conduct the statistical analysis. Statistical significance was set at a two-sided *p<*0.05 except for where a certain *p* ***-*** value has been given.

## Results

### Identification of Hub HLA Genes and Construction of an HLA Classifier

The flow chart of the dataset selection procedure is shown in Supplementary Figure S1.

**FIGURE 1:**
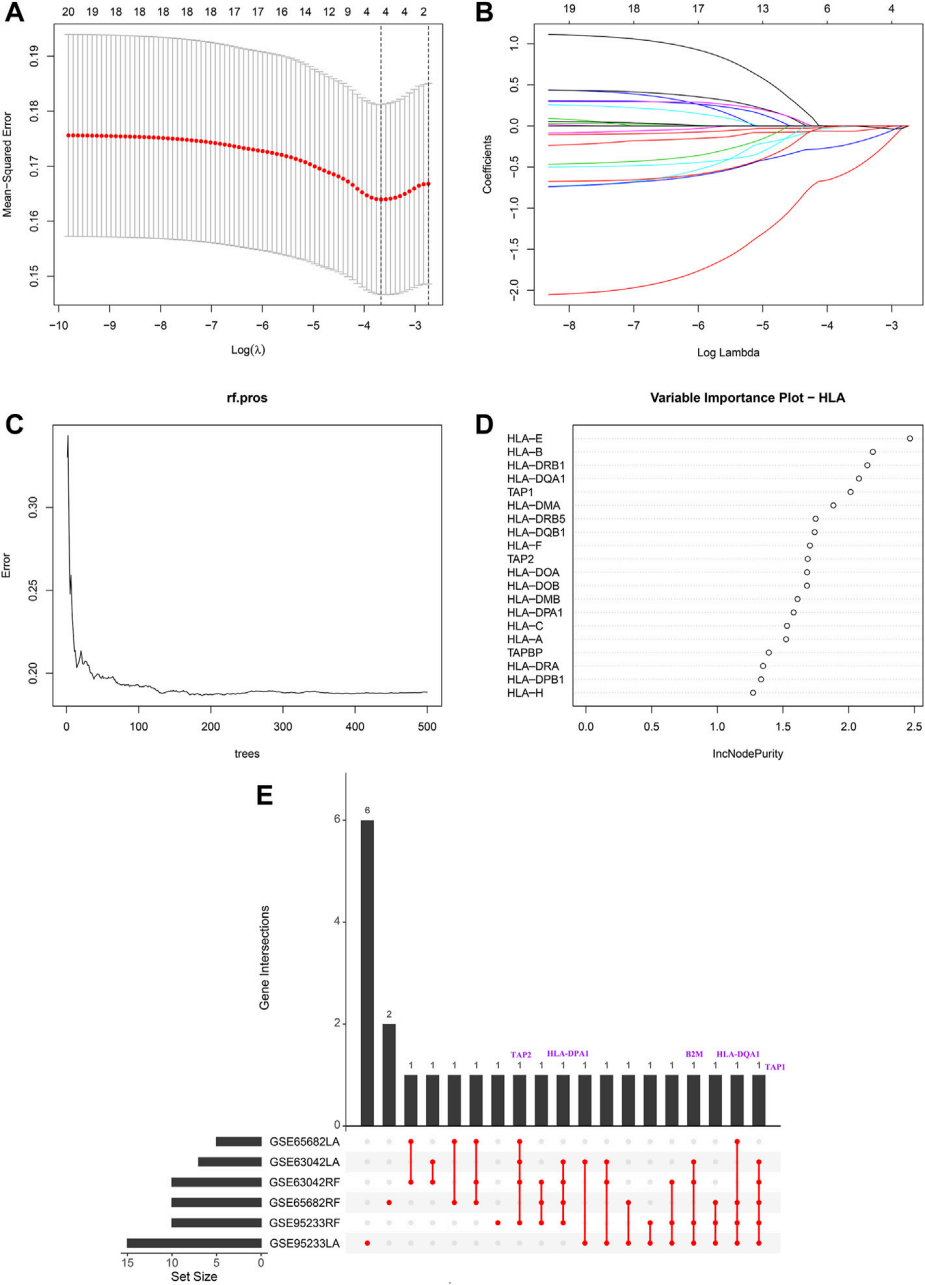
Hub HLA genes selected by Lasso regression analysis and random forest (RF) in GSE65682 datasets. **(A)** The two dotted vertical lines are drawn at the optimal values by minimum criteria (left) and 1—s.e. criteria (right). **(B)** Lasso coefficient profiles of the four hub HLA genes. A vertical line is drawn at the optimal value by minimum criteria and results in four non-zero coefficients. **(C)** Distribution diagram of regression tree and error. **(D)** Important variables ranked by IncNodePurity. **(E)** UpSet plot presents the intersection of six results to identify hub HLA genes.

Modified Lasso penalized regression was established to shrink and select out hub HLA genes in the discovery cohort, as shown in Figures 1A,B (GSE65682 set), in Supplementary Figures S2A,B (GSE69528 set), and in Supplementary Figures S3A,B (GSE95233 set). Likewise, an RF was also built with minimum error regression trees for hub HLA genes in the discovery cohort, as displayed in Figures 1C,D (GSE65682 set), in Supplementary Figures S2C,D (GSE69528 set), and in Supplementary Figures S3C,D (GSE95233 set). According to the result of Lasso regression and RF in the discovery cohort, we took the intersection of 6 results to acquire 5 hub genes (B2M, HLA-DQA1, HLA-DPA1, TAP1, and TAP2) shared by ≥ 4 results (Figure 1E). Additionally, Supplementary Figure S4 displays the five hub HLA genes on the location of chromosomes. Finally, the five hub HLA genes were used to develop a prediction model, namely the HLA classifier, and the HLA score was computed.

### Diagnostic and Prognostic Value of the HLA Classifier

**FIGURE 2:**
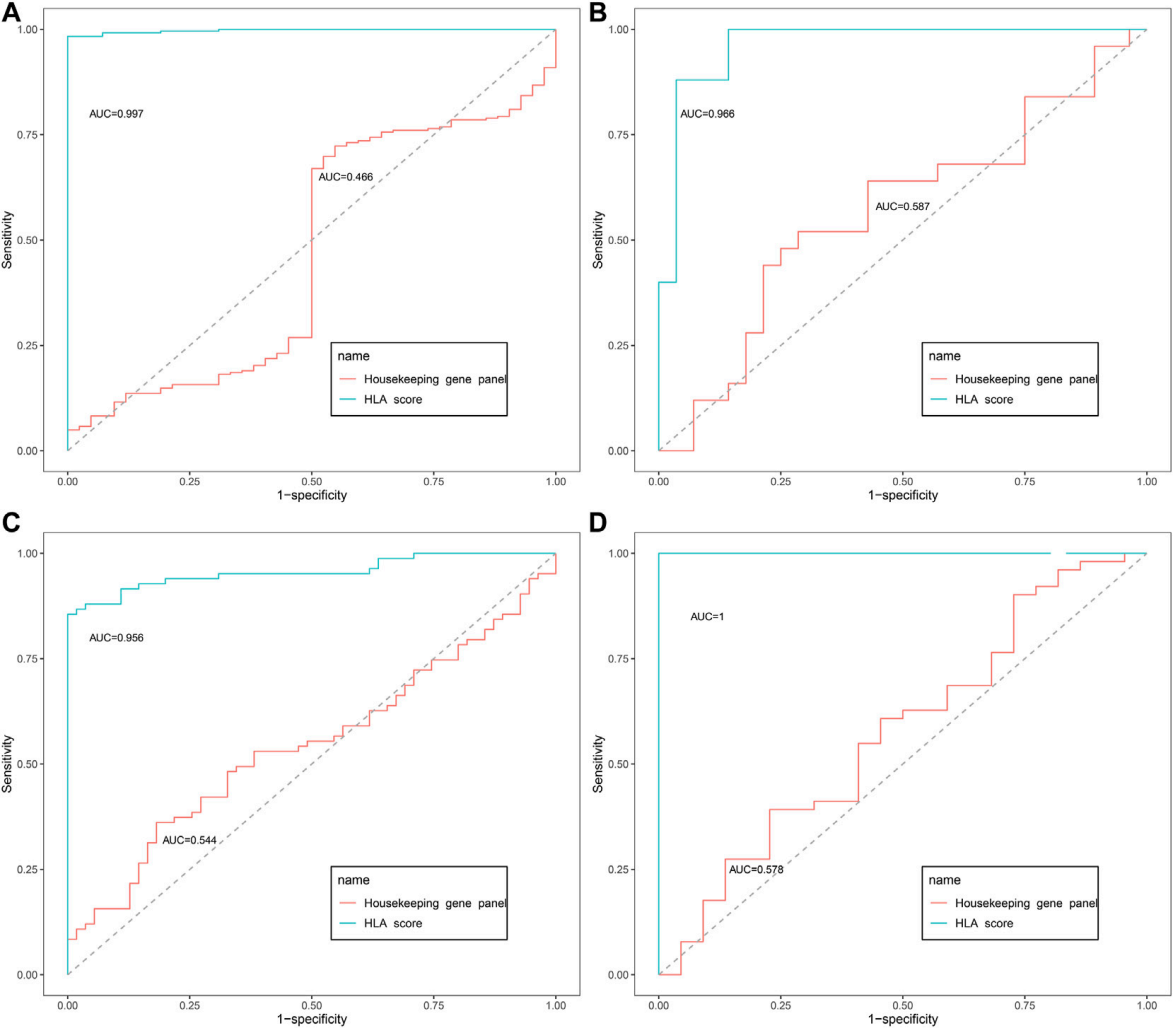
The diagnostic eﬃcacy of the HLA classifier in the discovery cohorts. **(A)** GSE65682 datasets. **(B)** GSE57065 datasets. **(C)** GSE69528 datasets. **(D)** GSE95233 datasets.

**FIGURE 3:**
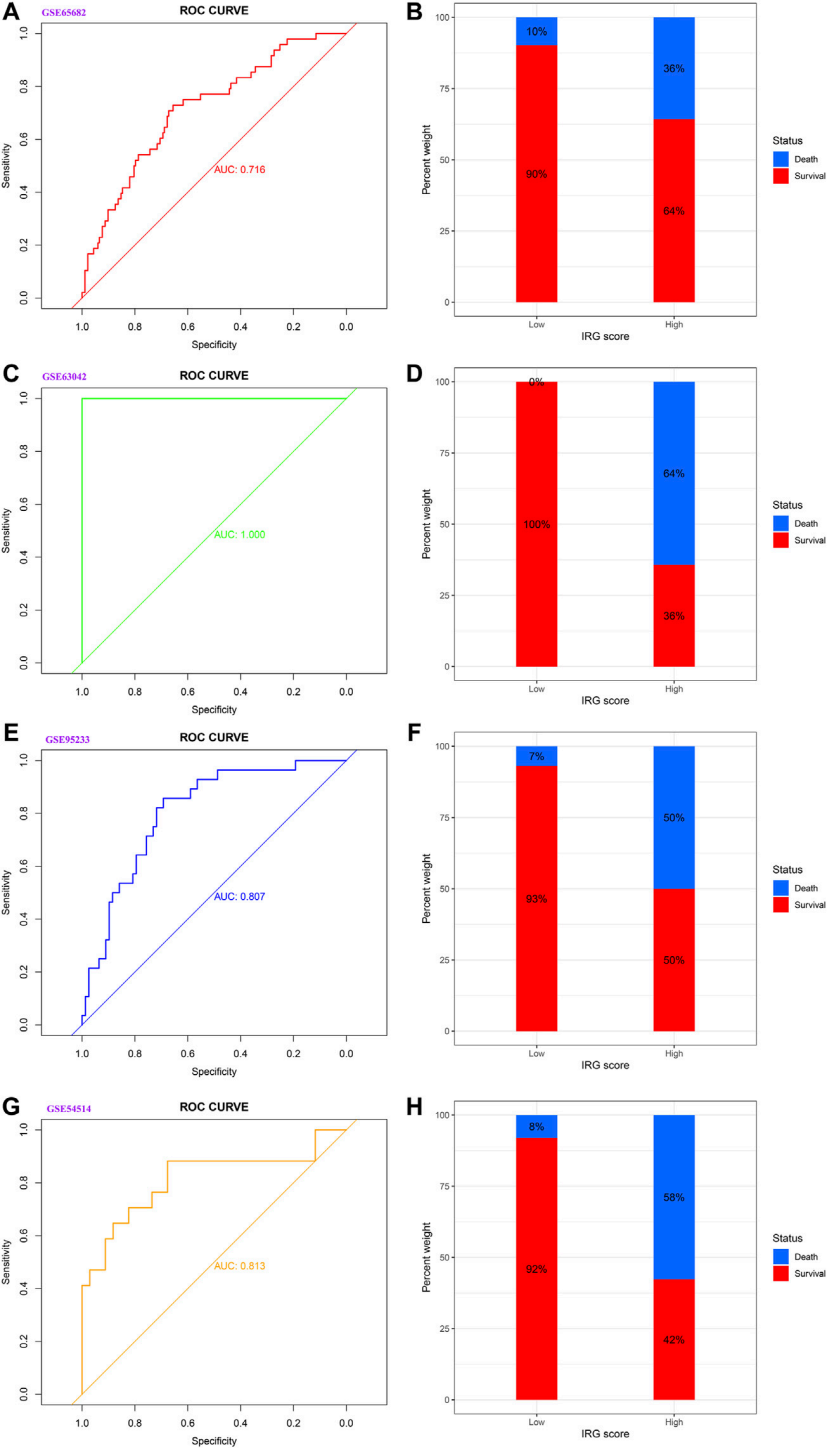
The prognostic capacity of the HLA classifier and the distribution of mortality rate in different HLA subgroups in the discovery cohorts. **(A,B)** GSE65682 datasets. **(C,D)** GSE63042 datasets. **(E,F)** GSE95233 datasets. **(G,H)** GSE54514 datasets.

**FIGURE 4:**
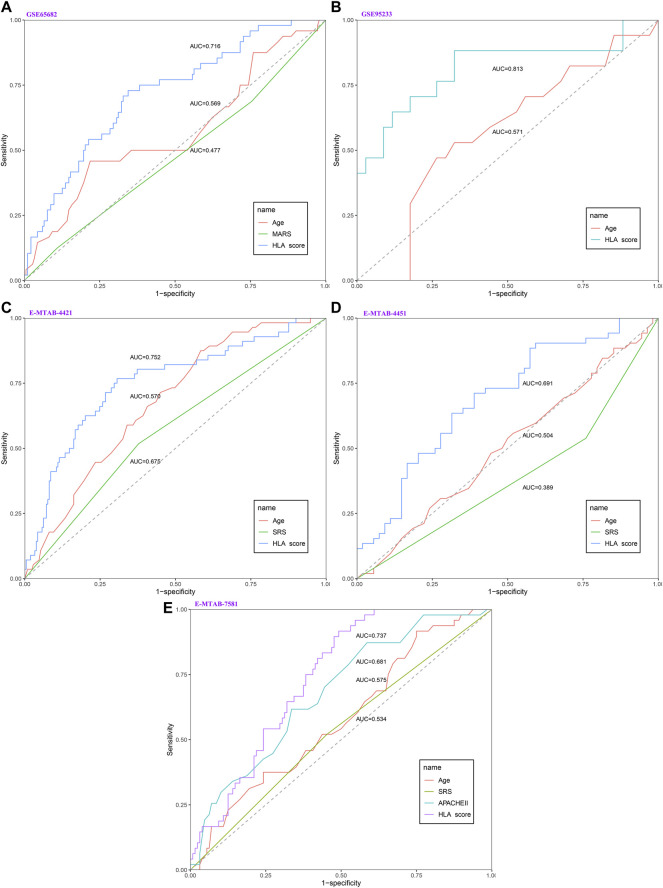
The evaluation of the performance of the HLA classifier compared against age, SRS, MARS, and APACHE II in the discovery and external validation cohorts. **(A)** GSE65682 datasets. **(B)** GSE95233 datasets. **(C)** E-MTAB-4421 datasets. **(D)** E-MTAB-4451 datasets. **(E)** E-MTAB-7581 datasets.

As displayed in Figure 2, the diagnostic ability of the HLA classifier to distinguish sepsis from the control samples showed superior diagnostic efficiency, with an AUC of 0.997 in the GSE65682 datasets, an AUC of 0.966 in the GSE57065 datasets, an AUC of 0.956 in the GSE69528 datasets, and an AUC of 1 in the GSE95233 datasets. However, the diagnostic ability of the housekeeping gene panel displayed inferior diagnostic efficiency for sepsis, with an AUC less than 0.6. As for prognostic value, univariate and multivariate Cox regression analysis confirmed that the HLA score was an independent predictor of unfavorable survival outcome, regardless of other clinical characteristics, in multiple transcriptome datasets (Table 2). In addition, as shown in Figure 3, ROC analysis was performed to investigate the prognostic value of the HLA classifier in the discovery cohorts, with an AUC of 0.716 in the GSE65682 datasets, an AUC of 0.807 in the GSE63042 datasets, an AUC of 0.813 in the GSE95233 datasets, and an AUC of 1 in the GSE54514 datasets. Similarly, the HLA classifier showed a favorable prognostic ability in the external validation cohort, with an AUC of 0.752 in the E-MTAB-4421 datasets, an AUC of 0.691 in the E-MTAB-4451 datasets, and an AUC of 0.737 in the E-MTAB-7851 datasets (Supplementary Figure S5). Based on the optimal cut-off value from the ROC curve, patients were categorized into the low-risk group (*n* = 133) or high-risk group (*n* = 98) in the GSE65682 sets, low-risk group (*n* = 21) or high-risk group (*n* = 14) in the GSE54514 sets, low-risk group (*n* = 58) or high-risk group (*n* = 48) in the GSE63042 sets, low-risk group (*n* = 25) or high-risk group (*n* = 26) in the GSE95233 sets, low-risk group (*n* = 140) or high-risk group (*n* = 125) in the E-MTAB-4421 sets, low-risk group (*n* = 29) or high-risk group (*n* = 77) in the E-MTAB-4451 sets, and low-risk group (*n* = 69) or high-risk group (*n* = 107) in the E-MTAB-4421 sets. Patients in the high-risk group showed a significantly higher mortality rate than in the low-risk group (*p* <0.001 for Chi-square test) in multiple transcriptome datasets (Figure 3 and Supplementary Figure S5). Importantly, the HLA classifier (AUC: 0.716) performed better in predicting mortality than age (AUC: 0.569) and MARS endotypes (AUC: 0.477) in the GSE65682 datasets, the HLA classifier (AUC: 0.813) performed better in predicting mortality than age (AUC: 0.571) in the GSE95233 datasets, the HLA classifier (AUC: 0.752) performed better in predicting mortality than SRS endotypes (AUC: 0.570) and age (AUC: 0.675) in the E-MTAB-4421 datasets, the HLA classifier (AUC: 0.691) performed better in predicting mortality than age (AUC: 0.504) and SRS endotypes (AUC: 0.390) in the E-MTAB-4451 datasets, and the HLA classifier (AUC: 0.737) performed better in predicting mortality than age (AUC: 0.575), SRS endotypes (AUC: 0.534), and APACHE II score (AUC: 0.681) in the E-MTAB-7851 datasets (Figure 4).

**TABLE 2:** Univariable and multivariable logistic regression analysis for prediction of survival in GEO and ArrayExpress databases.

Dataset	Factors	Subgroup	Univariable analysis		Multivariable analysis	
			OR (95%CI)	*p*	OR (95%CI)	*p*
GSE65682	—	—	—	—	—	—
	Age	—	1.02 (0.99–1.04)	0.114	NA	NA
	Sex	Female	1	—	—	—
		Male	1.42 (0.75–2.67)	0.284	NA	NA
	MARS	1–2	1	—	—	—
	—	3–4	0.93 (0.67–1.31)	0.688	NA	NA
	DM	No	1	—	—	—
	—	Yes	1.63 (0.71–3.74)	0.248	NA	NA
	HLA score	—	5.13 (2.03–15.39)	<0.001*	5.13 (2.03–15.39)	<0.001*
GSE63042	—	—	—	—	—	—
	HLA score	—	1.35 (4.22–43.18)	<0.001*	1.35 (4.22–43.18)	<0.001*
GSE54514	—	—	—	—	—	—
	HLA score	—	1.19 (1.06–1.45)	0.006*	1.19 (1.06–1.45)	0.006*
GSE95233	—	—	—	—	—	—
	Age	—	0.99 (0.95–1.03)	0.657	NA	NA
	Sex	Female	1	—	—	—
	—	Male	0.65 (0.35–1.18)	0.156	NA	NA
	HLA score	—	15.68 (3.04–80.93)	<0.001*	15.68 (3.04–80.93)	<0.001*
E-MTAB-4421	—	—	—	—	—	—
	Age	—	1.05 (1.02–1.07)	<0.001*	1.05 (1.03–1.08)	<0.001*
	Sex	Female	1	—	—	—
	—	Male	1.16 (0.64–2.10)	0.620	NA	NA
	SRS	1	—	—	—	—
	—	2	1.77 (0.98–3.20)	0.060	NA	NA
	HLA score	—	2.68 (1.44–4.97)	0.002*	2.93 (1.53–5.58)	0.001*
E-MTAB-4451	—	—	—	—	—	—
	Age	—	1.00 (0.98–1.03)	0.934	NA	NA
	Sex	Female	1	—	—	—
	—	Male	0.95 (0.40–2.28)	0.913	NA	NA
	SRS	1	—	—	—	—
	—	2	2.70 (1.18–6.19)	0.019	1.35 (0.50–3.70)	0.555
	HLA score	—	5.69 (2.08–15.58)	0.001*	5.69 (2.08–15.58)	0.001*
E-MTAB-7581	—	—	—	—	—	—
	Age	—	1.02 (0.99–1.04)	0.091	NA	NA
	Sex	Female	1	—	—	—
	—	Male	1.39 (0.71–2.72)	0.343	NA	NA
	SRS	1	—	—	—	—
	—	2	1.31 (0.68–2.55)	0.423	NA	NA
	APACHE II	—	1.08 (1.04–1.13)	0.001*	1.24 (1.06–1.19)	0.001*
	HLA score	—	11.35 (3.85–33.44)	<0.001*	17.74 (5.37–58.66)	<0.001*

Abbreviations: OR, odds ratio; CI, confidence intervals; MARS, the Molecular Diagnosis and Risk Stratification of Sepsis; DM, diabetes mellitus, APACHE II, acute physiology and chronic health evaluation; SRS, sepsis response signature. NOTE: NA, not available. These variables were eliminated in the multivariate logistic regression model, so the HR, and *p* values were not available. *p < 0.05.

### Clinical Usefulness of the HLA Classifier

**FIGURE 5:**
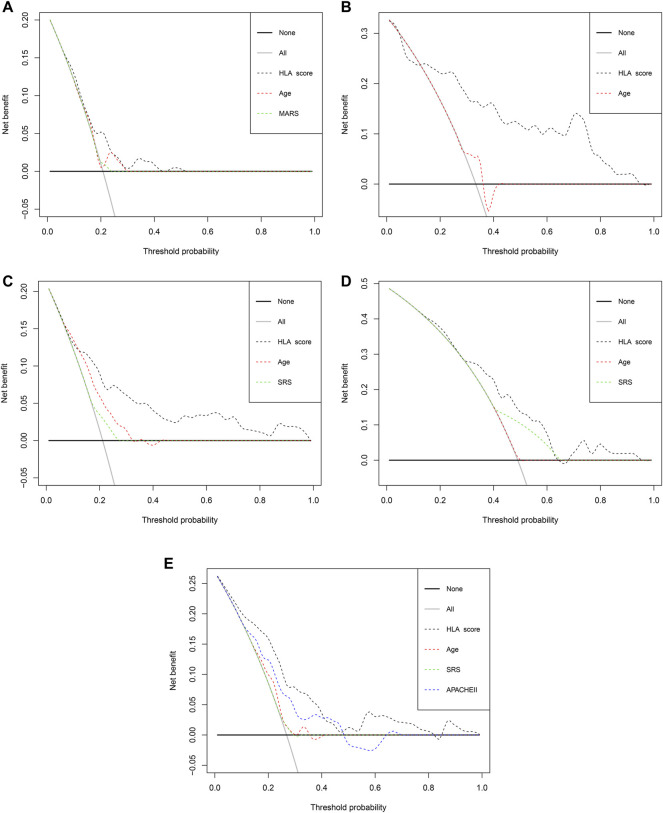
The evaluation of the clinical usefulness of the HLA classifier compared with age, SRS, MARS, and APACHE II in the discovery and external validation cohorts. **(A)** GSE65682 datasets. **(B)** GSE95233 datasets. **(C)** E-MTAB-4421 datasets. **(D)** E-MTAB-4451 datasets. **(E)** E-MTAB-7581 datasets.

The E-MTAB-7581 dataset was collected from the VANISH randomized trial with patients randomized to receive either vasopressin or norepinephrine followed by placebo or hydrocortisone. In the HLA high-risk subgroup, the use of hydrocortisone (OR: 2.84, 95% CI 1.07–7.57, *p* = 0.037) was associated with increased risk of mortality. In the HLA low-risk subgroup, the use of hydrocortisone (OR: 1.63, 95% CI 0.21–12.71, *p* = 0.644) did not render significant alteration (Table 3). Notably, the DCA chart showed that the HLA classifier outperformed age, SRS, MARS, and APACHE II according to the net benefit of risk stratification using the model (y-axis) and the continuity of potential death threshold (x-axis) in the discovery and external validation cohorts (Figure 5 and Supplementary Figure S6).

**TABLE 3:** Comparisons of the predictive value of the HLA classifier versus disease severity and SRS.

Models	Univariable analysis	
	OR (95% CI)	*p*
Use of hydrocortisone in the HLA low-risk subgroup	1.63 (0.21–12.71)	0.644
Use of hydrocortisone in the HLA high-risk subgroup	2.84 (1.07–7.57)	0.037*
Use of hydrocortisone in SRS 2	3.76 (1.41–10.04)	0.008*
Use of hydrocortisone in SRS 1	1.25 (0.47–3.36)	0.658
Use of hydrocortisone by APACHE II	0.94 (0.86–1.03)	0.210
Use of vasopressin in SRS 2	0.69 (0.18–2.62)	0.583
Use of vasopressin in SRS 1	1.50 (0.40–3.89)	0.403
Use of vasopressin in the HLA low-risk subgroup	2.91 (0.29–29.45)	0.366
Use of vasopressin in the HLA high-risk subgroup	1.26 (0.58–2.76)	0.559
Use of vasopressin by APACHE II	0.96 (0.87–1.06)	0.427

The logistic regression models integrating interactions between treatment allocation and SRS, HLA, classifier, or APACHE II, were built in the E-MTAB-7581, dataset. A total of six logistic regression models were built by using mortality as the response variable and respective predictors and interactions were: hydrocortisone & class, hydrocortisone and SRS, hydrocortisone and APACHE II, vasopressin and class, vasopressin and SRS, and vasopressin and APACHE II. A signiﬁcant (***p*** <0.05) interaction indicated that the classiﬁcation method was of predictive value because it identiﬁed that a subgroup of patients responded differently to treatment. SRS, classiﬁcation was used as previously reported. Abbreviations: OR, odds ratio; CI, confidence intervals; MARS, the Molecular Diagnosis and Risk Stratification of Sepsis; DM, diabetes mellitus, APACHE II, acute physiology and chronic health evaluation; SRS, sepsis response signature.

### Immune Cell Infiltration Analysis

**FIGURE 6:**
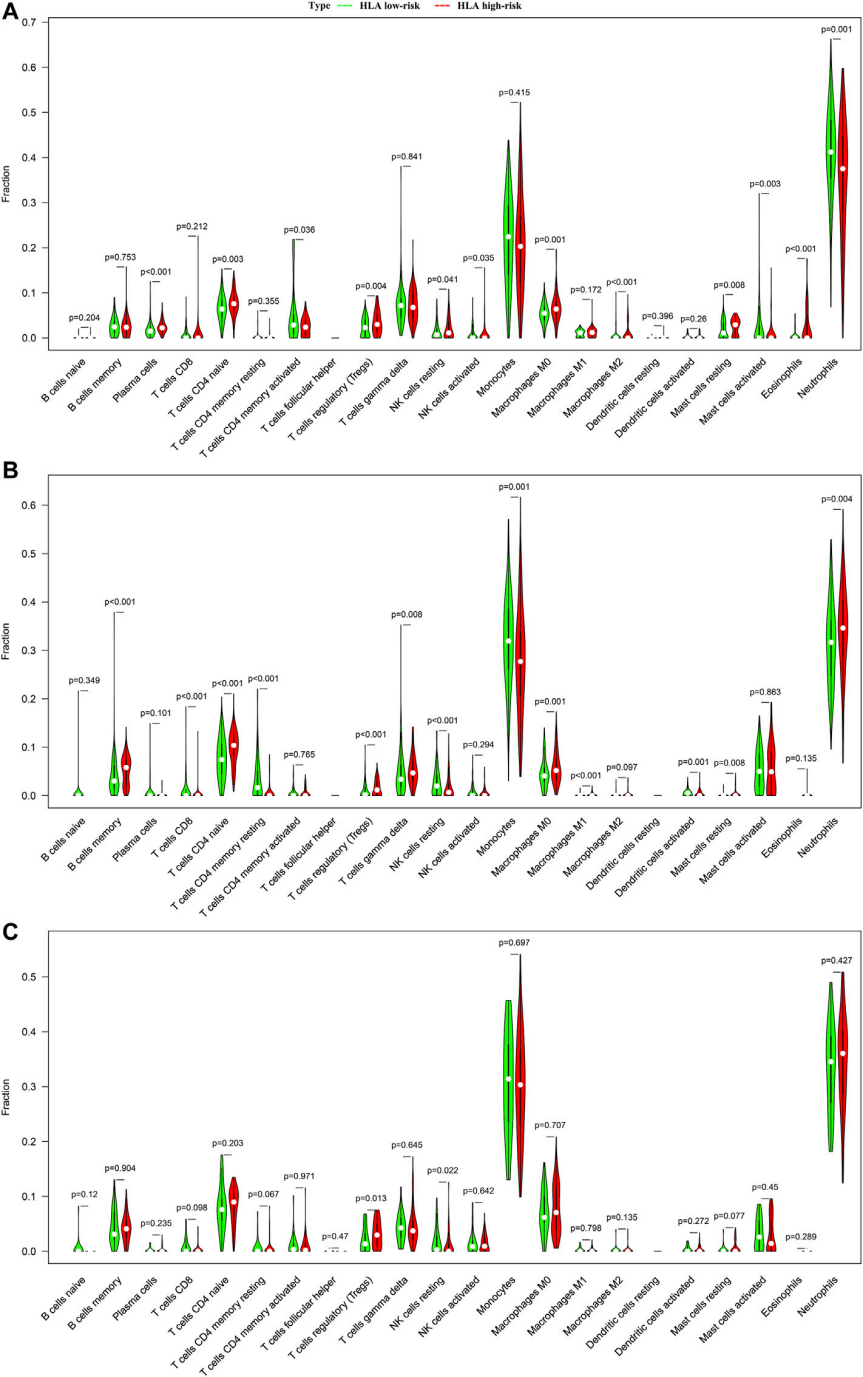
Comparison of infiltrating immune cells between different HLA subgroups based on CIBERSORTx in multiple transcriptome datasets. **(A)** GSE65682 datasets. **(B)** E-MTAB-4421 datasets. **(C)** E-MTAB-4451 datasets. Green indicates HLA low-risk, while red indicates HLA high-risk.

We analyzed the difference in composition of immune cells between the HLA subgroups in multiple transcriptome sets. The CIBERSORTx results demonstrated that compared with the HLA high-risk subgroup, activated memory CD4 T cells (*p* = 0.036), activated NK cells (*p* = 0.035), neutrophils (*p* = 0.001), and activated mast cells (*p* = 0.003) were more abundant in the HLA low-risk subgroup, while naive CD4 T cells (*p* = 0.003), regulatory T cells (Tregs) (*p* = 0.004), M0 macrophages (*p* = 0.001), and resting mast cells (*p* = 0.008) were more abundant in the high-risk subgroup than in the low-risk subgroup (Figure 6A) in GSE65682 datasets. In E-MTAB-4421 datasets, CD8 T cells (*p* < 0.001), resting memory CD4 T cells (*p* < 0.001), resting NK cells (*p* < 0.001), monocytes (*p* < 0.001), and activated dendritic cells (*p* = 0.001) were more abundant in the low-risk subgroup, but memory B cells (*p* < 0.001), naive CD4 T cells (*p* < 0.001), Tregs (*p* < 0.001), and M0 macrophages (*p* = 0.001) were more abundant in the high-risk subgroup than in the low-risk subgroup (Figure 6B). In E-MTAB-4451 datasets, the CIBERSORTx results uncovered that compared to the high-risk group, resting NK cells (*p* = 0.002) were more abundant in the low-risk subgroup, yet Tregs (*p* = 0.005) were more abundant in the high-risk subgroup than in the low-risk subgroup (Figure 6C). Supplementary Figure S7, Supplementary Figure S8, and Supplementary Figure S9 display the distribution of 22 types of immune cells in each sample for GSE65682 datasets, E-MTAB-4421 datasets, and E-MTAB-4451 datasets, respectively. In total, NK cells were significantly enriched in the HLA low-risk subgroup, whereas Tregs were more abundant in the HLA high-risk subgroup.

**FIGURE 7:**
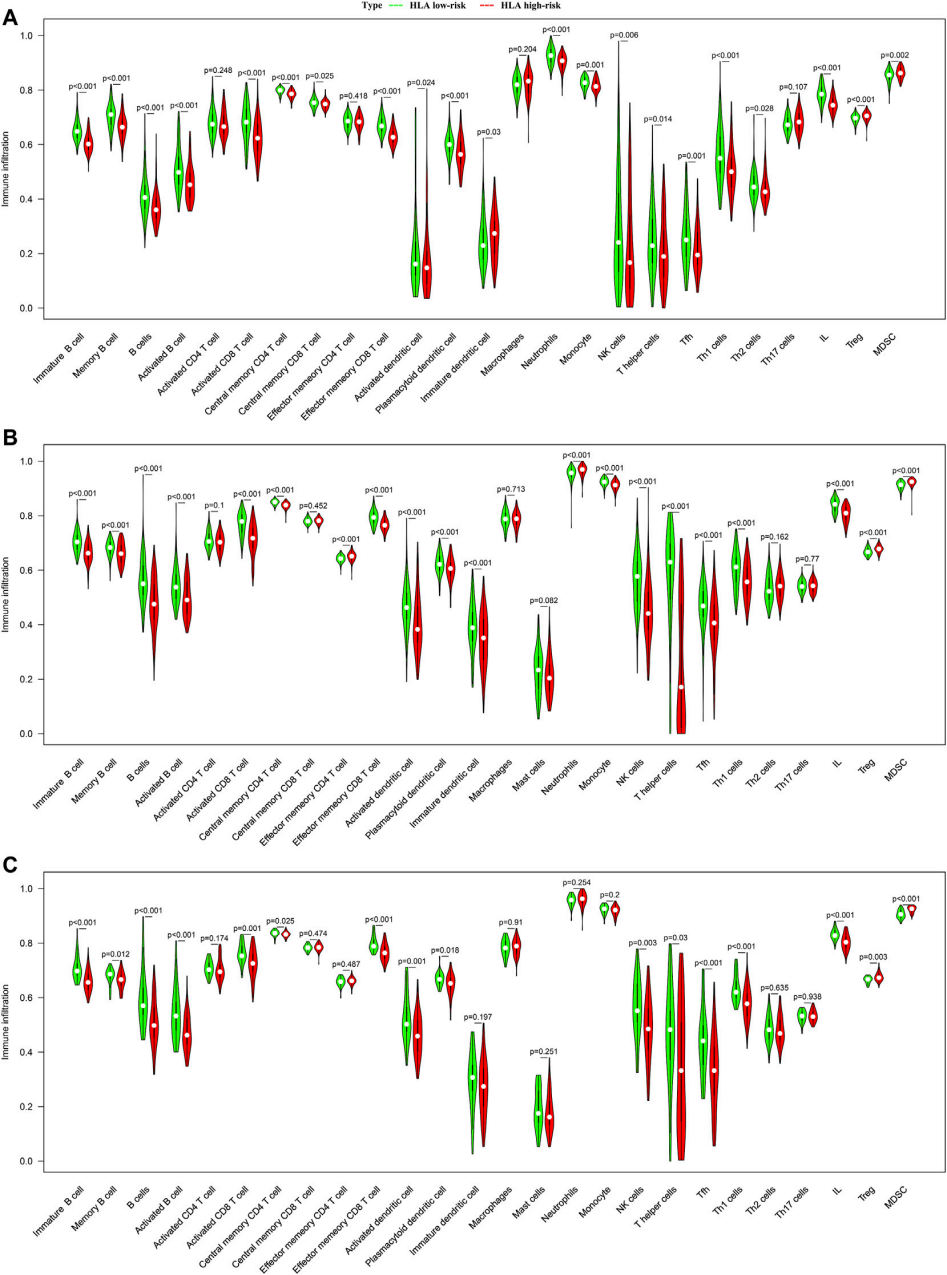
Comparison of infiltrating immune cells between different HLA subgroups based on ssGSEA in multiple transcriptome datasets. **(A)** GSE65682 datasets. **(B)** E-MTAB-4421 datasets. **(C)** E-MTAB-4451 datasets. Green indicates HLA low-risk, while red indicates HLA high-risk.

In addition, we adopted ssGSEA, another cell-type quantification method, to quantify the enrichment score of immune cell types. Compared to the CIBERSORTx results, the ssGSEA results revealed that significant infiltration of immune cells was concentrated in the HLA low-risk subgroup. In GSE65682 datasets, compared with the high-risk subgroup, activated B cells (*p* < 0.001), activated CD8 T cells (*p* < 0.001), NK cells (*p* < 0.001), activated dendritic cells (*p* = 0.024), T helper cells (*p* < 0.001), and infiltrating lymphocytes (IL) (*p* < 0.001) were more abundant in the low-risk subgroup, whereas Tregs (*p* < 0.001) and myeloid-derived suppressor cells (MDSCs) (*p* = 0.002) were more enriched in the high-risk subgroup than in the low-risk subgroup (Figure 7A). Similar results were observed in E-MTAB-4421 (Figure 7B) and E-MTAB-4451 datasets (Figure 7C), which indicated that patients in the HLA high-risk subgroup were characterized by immunosuppression.

### Correlation Between HLA Classifier/Genes and Immune Cells

**FIGURE 8:**
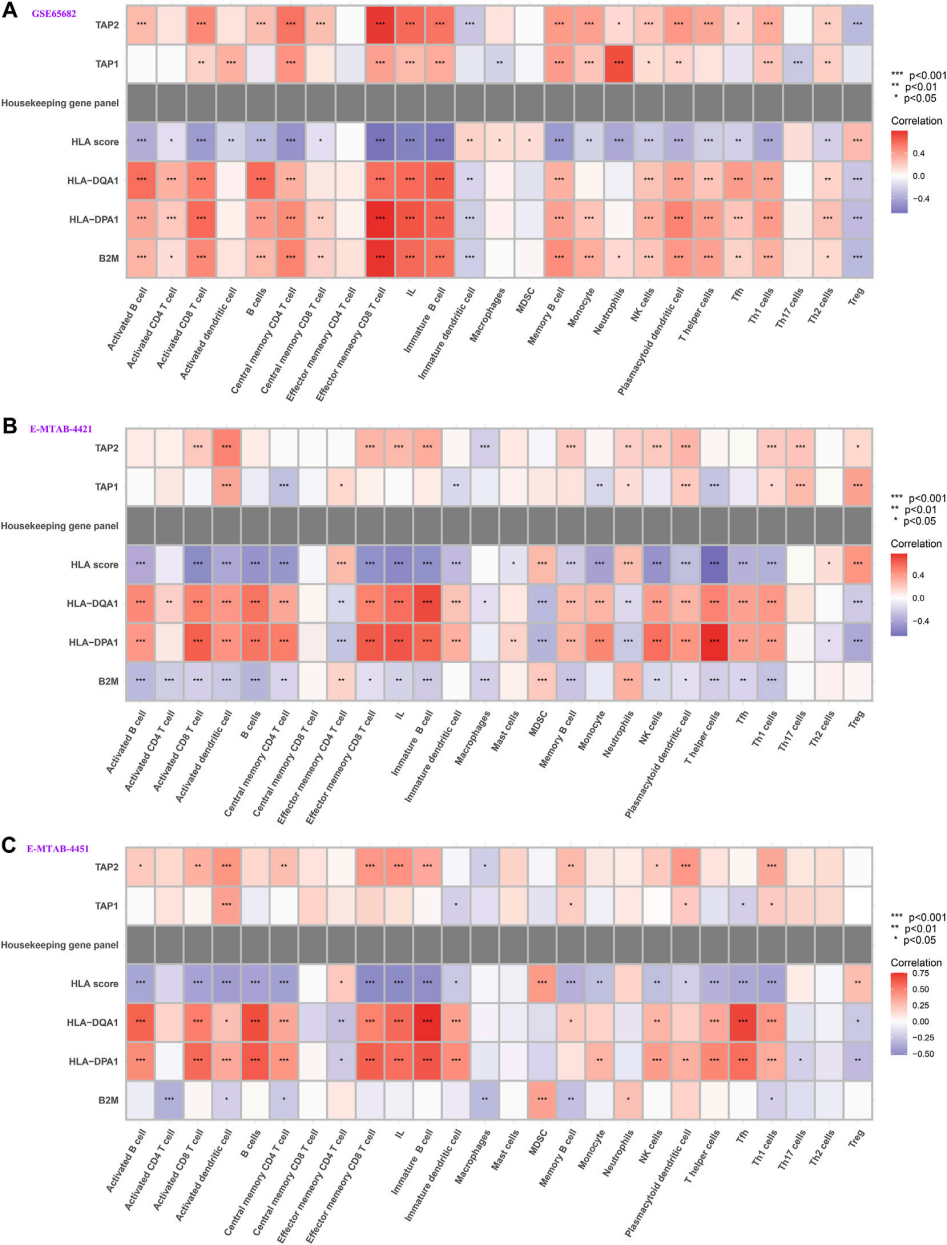
Correlation between HLA classifier/genes and immune cells in multiple transcriptome datasets. **(A)** GSE65682 datasets. **(B)** E-MTAB-4421 datasets. **(C)** E-MTAB-4451 datasets.

We further explored whether our HLA classifier/genes were related to immune cell infiltration in sepsis via Spearman correlation analyses in multiple gene expression profiles. In the GSE65682 dataset, the HLA score was significantly negatively correlated with B cells (*p* < 0.001), NK cells (*p* < 0.001), activated dendritic cells (*p*<0.01), ILs (*p* < 0.001), and T helper cells (*p* < 0.001), whereas the HLA score was significantly positively correlated with Tregs (*p* < 0.001) and MDSCs (*p* < 0.05) (Figure 8A and Supplementary Table S5). Likewise, in the E-MTAB-4421 dataset, the HLA score was significantly negatively correlated with B cells (*p* < 0.001), NK cells (*p* < 0.001), activated dendritic cells (*p* <0.001), ILs (*p* < 0.001), and T helper cells (*p* < 0.001), whereas the HLA score was significantly positively correlated with Tregs (*p* < 0.001) and MDSCs (*p* < 0.001) (Figure 8B and Supplementary Table S6). Similarly, in the E-MTAB-4451 dataset, the HLA score was significantly negatively correlated with B cells (*p* < 0.001), NK cells (*p* < 0.01), activated dendritic cells (*p* < 0.001), ILs (*p* < 0.001), and T helper cells (*p* < 0.001), whereas the HLA score was significantly positively correlated with Tregs (*p* < 0.01) and MDSCs (*p* < 0.001) (Figure 8C and Supplementary Table S7). In addition, Figure 8 shows that B2M, HLA-DQA1, HLA-DPA1, TAP1, and TAP2 were significantly associated with the infiltration of immune cells, HLA-DPA1 and HLA-DQA1 in particular. Nevertheless, the housekeeping gene panel is not correlated with immune cell infiltration (Figure 8).

### Immune and Molecular Function Between the HLA Subgroups by GSVA and ssGSEA

To screen biological diﬀerences between the HLA subgroups, GSVA was conducted to determine the gene sets enriched in different HLA subgroups. In GSE65682 sets (Supplementary Figure S10A), the results showed that the NOD-like receptor signaling pathway, toll-like receptor signaling pathway, and complement and coagulation cascades were enriched in the HLA high-risk group, yet alanine aspartate and glutamate metabolism and glyoxylate and dicarboxylate metabolism were mainly involved in the HLA low-risk group. In GSE95233 sets (Supplementary Figure S10B), primary immunodeficiency, the PPAR signaling pathway, and complement and coagulation cascades were mainly enriched in the HLA high-risk group, but antigen processing and presentation and aminoacyl tRNA biosynthesis were involved in the HLA low-risk subgroup. In E-MTAB-4421 sets (Supplementary Figure S10C), spliceosome, valine leucine, and isoleucine degradation and RNA degradation were enriched in the HLA high-risk subgroup, while starch and sucrose metabolism, leukocyte transendothelial migration, and neuroactive ligand receptor interaction were mainly involved in the HLA low-risk subgroup. To sum up, complement and coagulation cascades may play an important role in the initiation and progression of sepsis.

**FIGURE 9:**
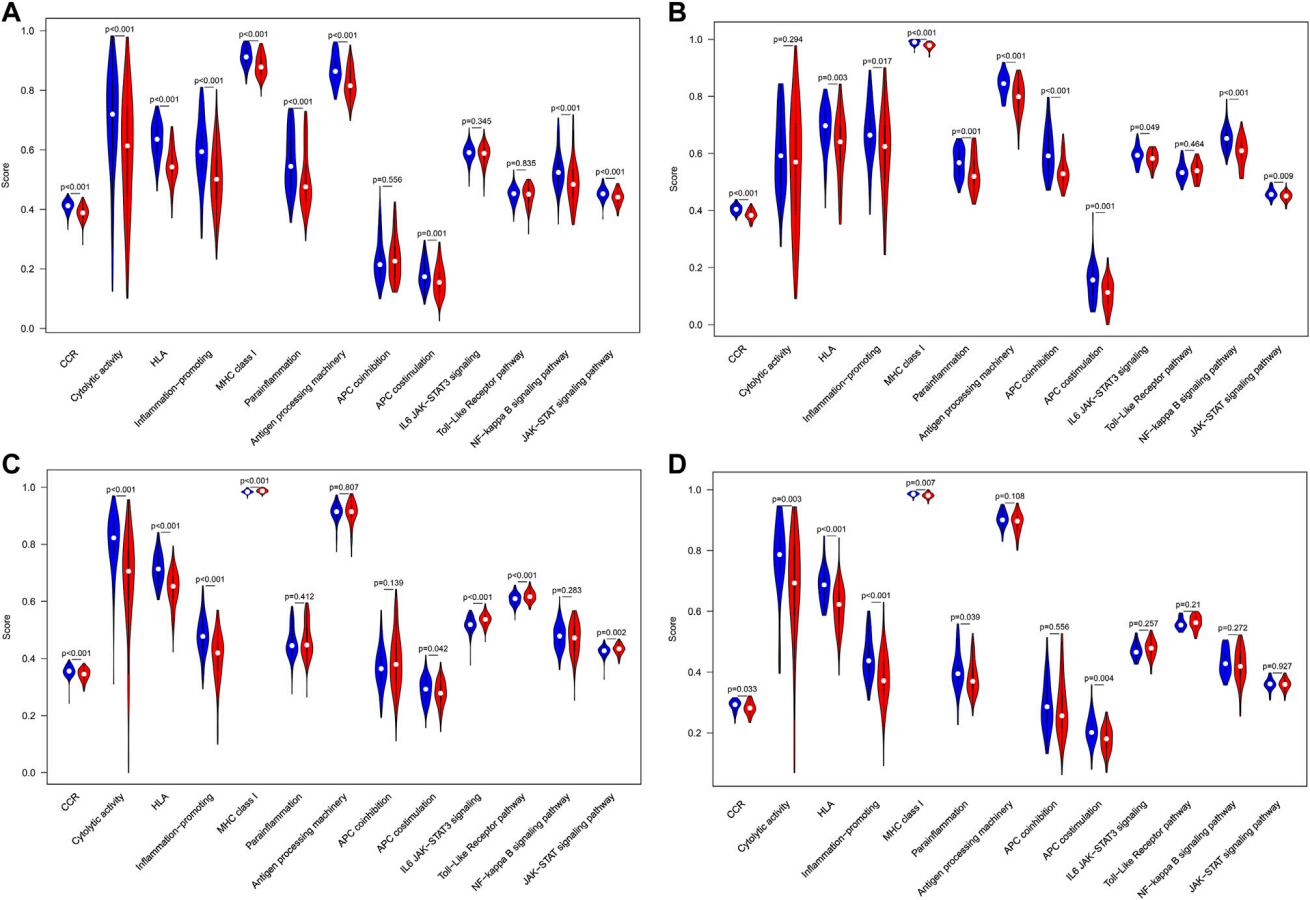
Comparison of immune-related pathways between different HLA subgroups in the discovery cohorts. **(A)** GSE65682 datasets. **(B)** GSE63042 datasets. **(C)** E-MTAB-4421 datasets. **(D)** E-MTAB-4451 datasets. Blue indicates HLA low-risk, while red indicates HLA high-risk.

In addition, ssGSEA was utilized to investigate the given immune-related pathway in sepsis. As a result, in GSE65682 datasets, all of the significantly different immune-related gene sets were enriched in the HLA low-risk subgroup, such as cytokine-cytokine receptor (CCR) interaction, cytolytic activity, human leukocyte antigen (HLA), inflammation−promoting, MHC class I, antigen processing machinery, antigen-presenting cell (APC) costimulation, parainflammation, the NF−kappa B signaling pathway, and the JAK−STAT signaling pathway (Figure 9A). Analogously, in GSE63042 datasets, the gene sets of the HLA low-risk group were enriched in CCR, HLA, inflammation−promoting, antigen processing machinery, APC coinhibition, APC costimulation, parainflammation, IL6 JAK−STAT3 signaling, the NF−kappa B signaling pathway, and the JAK−STAT signaling pathway (Figure 9B). Homoplastically, in E-MTAB-4421 datasets, the ssGSEA results showed that CCR, cytolytic activity, HLA, inflammation-promoting, and APC costimulation were involved in the HLA low-risk group (Figure 9C). Similarly, in E-MTAB-4451 datasets, the ssGSEA results demonstrated that CCR, cytolytic activity, HLA, inflammation-promoting, parainflammation, and APC costimulation were mainly enriched in the HLA low-risk group (Figure 9D). In short, the HLA high-risk group, compared with the HLA low-risk group, was characterized by immunosuppression in which many pivotal immune pathways were suppressed such as CCR, HLA, inflammation-promoting, and APC costimulation.

### Correlation Between HLA Classifier/Genes and Pivotal Molecular Pathways

**FIGURE 10:**
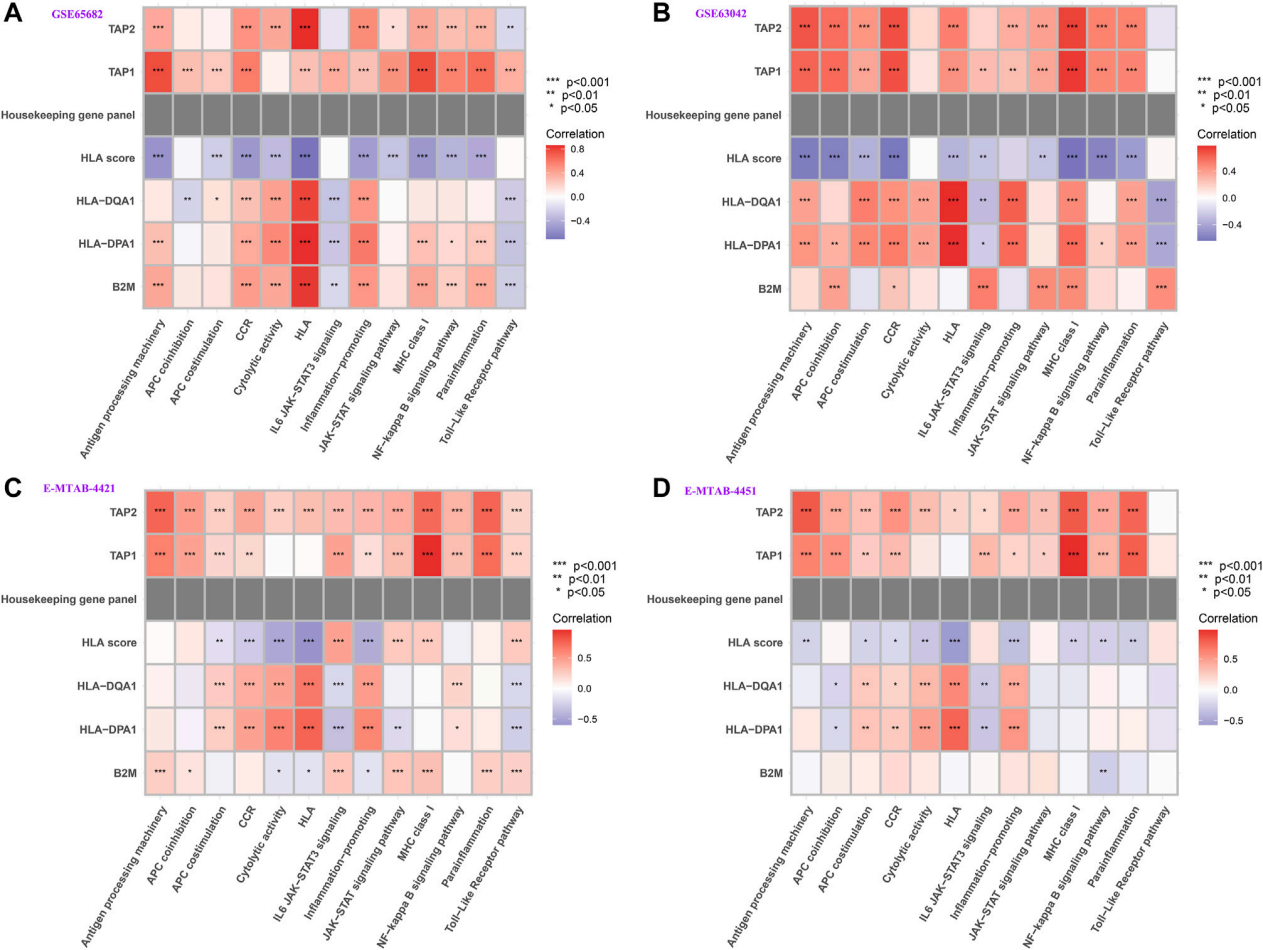
Correlation between HLA classifier/genes and immune-related pathways in multiple transcriptome datasets. **(A)** GSE65682 datasets. **(B)** GSE63042 datasets. **(C)** E-MTAB-4421 datasets. **(D)** E-MTAB-4451 datasets.

We further tested whether our HLA classifier/genes were related to molecular pathways in sepsis via Spearman correlation analyses in multiple transcriptome sets. Encouragingly, the HLA score was significantly negatively correlated with HLA (*p* <0.001), APC costimulation (*p* < 0.001), parainflammation (*p* < 0.001), antigen processing machinery (*p* < 0.001), and CCR (*p* < 0.001) in GSE65682 sets; HLA (*p* < 0.001), APC costimulation (*p* < 0.001), inflammation-promoting (*p* < 0.001), antigen processing machinery (*p* < 0.001), and CCR (*p* < 0.001) in GSE63042 sets; HLA (*p* < 0.001), APC costimulation (*p* < 0.001), inflammation-promoting (*p* < 0.001), MHC class I (*p* < 0.001), and CCR (*p* < 0.001) in E-MTAB-4421 sets; and HLA (*p* < 0.001), APC costimulation (*p* < 0.05), inflammation-promoting (*p* < 0.001), antigen processing machinery (*p* < 0.01), and CCR (*p* < 0.05) in E-MTAB-4451 sets (Figure 10). In addition, Figure 10 shows that B2M, HLA-DQA1, HLA-DPA1, TAP1, and TAP2 were significantly associated with the enrichment score of immune-related pathways, TAP1 and TAP2 in particular. Nevertheless, the housekeeping gene panel is not correlated with immune-related pathways (Figure 10).

### Analyses of the Cytokines

**FIGURE 11:**
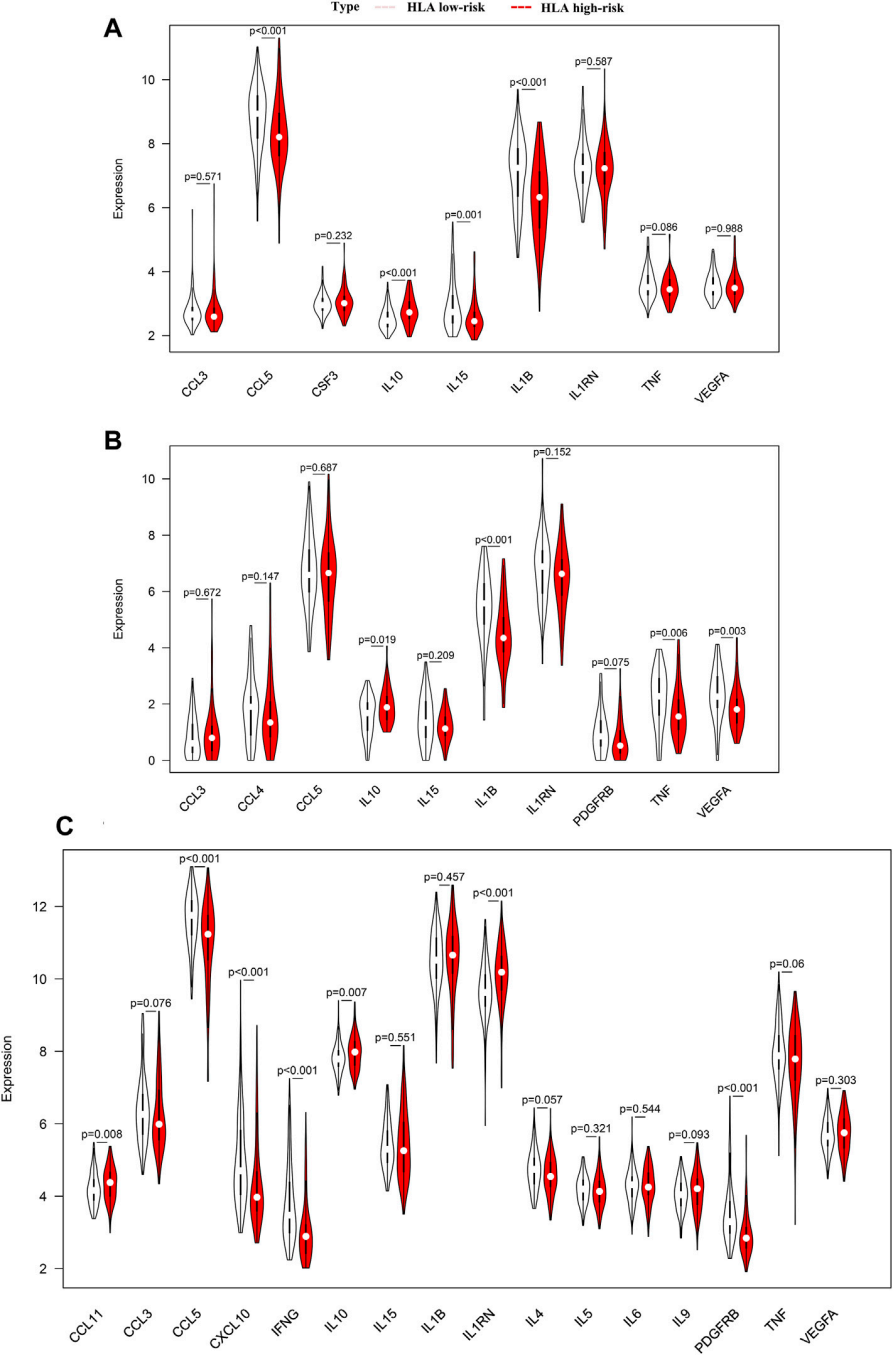
Comparison of the expression level of cytokines between different HLA subgroups in multiple transcriptome datasets. **(A)** GSE65682 datasets. **(B)** GSE63042 datasets. **(C)** E-MTAB-4421 datasets. Pink indicates HLA low-risk, while red indicates HLA high-risk.

To analyze the clinically detectable inflammatory cytokines involved in sepsis, we applied the Wilcoxon test to compare the expression levels of cytokines in different HLA endotypes. As a result, in GSE65682 sets, the expression levels of CCL5, IL1B, and IL15 were significantly higher in the HLA low-risk group, but the expression levels of IL10 were significantly downregulated in the HLA low-risk group (Figure 11A). Analogously, in GSE63042 sets, the expression levels of IL1B, TNF and VEGFA were significantly higher in the HLA low-risk group, but the expression levels of IL10 were significantly lower in the HLA low-risk group (Figure 11B). Similarly, in E-MTAB-4421 sets, the expression levels of CCL5, CXCL10, IFNG, and PDGFRB were significantly higher in the HLA low-risk group, and the expression levels of TNF exhibited a trend toward a higher expression in the HLA low-risk group, but the expression levels of CCL11, IL10, and IL1RN were significantly lower in the HLA low-risk group (Figure 11C). In summary, pro-inflammatory cytokines were upregulated in the HLA low-risk subgroup and anti-inflammatory cytokines were upregulated in the HLA high-risk subgroup.

**FIGURE 12:**
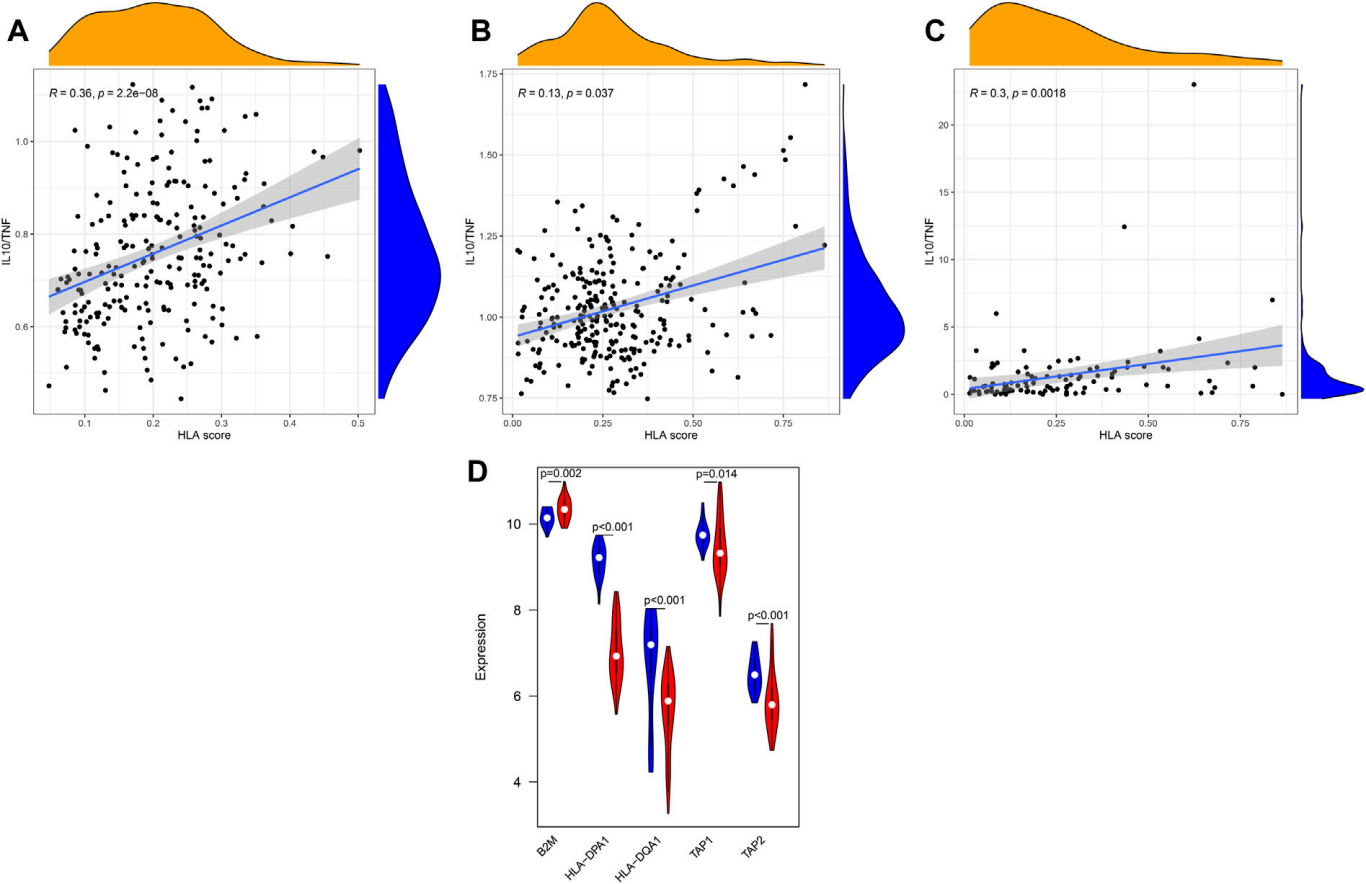
Correlation between the HLA classifier and the ratio of IL10/TNF in multiple transcriptome datasets. **(A)** GSE65682 datasets. **(B)** GSE63042 datasets. **(C)** E-MTAB-4421 datasets. **(D)** Comparison of gene expression levels of five HLA genes between controls and sepsis samples *via* qRT-PCR. Red indicates sepsis, while blue indicates normal.

In addition, we further explored whether our HLA classifier was associated with the ratio of IL10/TNF in sepsis. As a results, the HLA score was significantly positively correlated with IL10/TNF (R = 0.36, *p* < 0.001) in GSE65682 sets, positively correlated (R = 0.13, *p* = 0.037) in GSE63042 sets, and positively correlated (R = 0.3, *p* = 0.0018) in E-MTAB-4421 sets (Figures 12A–C).

### qRT-PCR

To further validate the expression of the five HLA genes, we performed qRT-PCR in 50 clinical specimens. Compared with healthy controls, B2M was significantly upregulated in sepsis samples (Figure 12D) yet the expression level of HLA-DQA1, HLA-DPA1, TAP1, and TAP2 was significantly lower in sepsis specimens. Collectively, the results of qRT-PCR were in accordance with the results of bioinformatics analyses derived from the GEO datasets.

## Discussion

After analyzing multiple gene expression profiling, according to modified Lasso penalized regression and RF, five HLA genes (B2M, HLA-DQA1, HLA-DPA1, TAP1, and TAP2) were identified as hub genes, which were used to construct a prediction model, namely the HLA classifier. In the discovery cohort, the HLA classifier exhibited superior diagnostic efficacy (AUC = 0.997) and performed better in predicting mortality (AUC = 0.716) than clinical characteristics or MARS/SRS endotypes. Encouragingly, similar results were observed in the ArrayExpress databases. In the E-MTAB-7581 dataset, the use of hydrocortisone in the HLA high-risk subgroup (OR: 2.84, 95% CI 1.07–7.57, *p* = 0.037) was associated with increased risk of mortality. Immune infiltration analysis by CIBERSORTx showed that NK cells were significantly enriched in the HLA low-risk subgroup, while Tregs were more abundant in the HLA high-risk subgroup. Intriguingly, ssGSEA also revealed that B cells, activated dendritic cells, NK cells, T helper cells, and ILs were significantly enriched in the HLA low-risk subgroup, while Tregs and MDSCs were more abundant in the HLA high-risk subgroup. The HLA score was significantly negatively correlated with the infiltration score of B cells, activated dendritic cells, NK cells, T helper cells, and ILs, yet was significantly positively correlated with the infiltration score of Tregs and MDSCs. Additionally, molecular pathways determined via the ssGSEA algorithm uncovered that CCR, HLA, and APC costimulation was significantly enriched in the HLA low-risk subgroup, enrichment scores of which were significantly negatively correlated with HLA score. Finally, the expression levels of several cytokines (IL-10, IFNG, TNF) were significantly different between the HLA phenotypes, and the ratio of IL-10/TNF was significantly positively correlated with HLA score. Results of qRT-PCR validated the higher expression level of B2M as well as lower expression level of HLA-DQA1, HLA-DPA1, TAP1, and TAP2 in sepsis samples compared to control samples.

To the best of our knowledge, this is the first comprehensive study to explore HLA gene sets based on a multiple transcriptome expression profiles in all-cause sepsis, leading to the discovery of novel biomarkers to develop a diagnostic and prognostic model, thus elucidating the model and immune system (immune cell infiltration, immune-related pathways, and cytokines) to find its additional clinical implications.

At present, no single biomarker can be efficient in diagnosing sepsis, prognosis, and monitoring disease with especially high performance uniformly according to the variety of factors and processes involved in sepsis (Jensen and Bouadma, 2016). This is most likely due to heterogeneity in the adult host response to infection and fails to capture important pathophysiological alterations, thus cannot uncover underlying mechanisms. HLA gene sets, as promising novel biomarkers, may offer important predictive and prognostic information. Machine learning methods, which can decrease diagnostic uncertainties and analyze the heterogeneity in transcriptome data (Baniasadi et al., 2021), including RF based on minimum error regression trees and modified Lasso coupled with adequate validation metrics, were applied to identify reliable feature variables. Based on RF and Lasso, five HLA genes (B2M, HLA-DQA1, HLA-DPA1, TAP1, and TAP2) were identified as hub genes, which were combined to construct an HLA classifier. As to diagnostic ability, the AUC of the HLA classifier was more than 0.95 in multiple transcriptome sets, which demonstrated that the HLA classifier can efficiently discriminate sepsis from the control samples. As for prognostic capacity, the HLA classifier was an independent predictor of unfavorable survival outcome, regardless of other clinical characteristics, in multiple transcriptome datasets. Importantly, the performance of the HLA classifier in predicting mortality outcomes was superior to clinical features or MARS/SRS endotypes. In total, the model, HLA classifier, could be a robust tool to diagnose sepsis earlier and to identify patients at risk of a poor or even fatal outcome.

Up to now, prognostic biomarkers/models have mainly been utilized for overall prognosis, which is not enough. Added information should include how to stratify patients to guide treatment. Interestingly, our results found that though the HLA classifier could not modify the effect of norepinephrine versus vasopressin, the HLA high-risk subgroup exhibited a significantly higher mortality outcome when assigned to the hydrocortisone group, consistent with the GAinS study where the use of hydrocortisone in SRS1, which represents an immunosuppressed phenotype including features of downregulation of HLA class II, endotoxin tolerance, and T-cell exhaustion, was associated with increased risk of mortality (Davenport et al., 2016). The probable explanation is that the HLA low-risk subgroup was relatively immunocompetent with a lower mortality rate, and the HLA high-risk subgroup was relatively immunocompromised with a higher mortality rate. The use of hydrocortisone suppresses the immune system (Steinhagen et al., 2020), which aggravates the immunosuppression status of the HLA high-risk subgroup, thereby increasing the mortality rate. The HLA high-risk subgroup may not be suitable for the application of hydrocortisone. Additionally, DCA results indicated that survival-associated treatment decisions for sepsis patients based on the HLA classifier had a net benefit compared to treatment decisions based on other clinical features or MARS/SRS endotypes, or treatment for all patients or none. To sum up, the current HLA classifier could be useful for clinicians to tailor survival-related treatment decisions.

Excessive immune activation and concurrent immunosuppression are central to the pathophysiology of sepsis. Immunosuppression results in a profound dysfunction in innate and adaptive immune responses, which mainly manifests as the depletion and exhaustion of lymphocytes, increased apoptosis of immune cells, the expansion of Treg cells and MDSCs, downregulation of activating cell-surface molecules (HLA-DR), and inhibitory proinflammatory cytokine release (Hotchkiss et al., 2013). It is becoming increasingly clear that most sepsis patients are not succumbing to an overwhelming pro-inflammatory response early on, but rather to immunoparalysis-related complications that occur later in the disease trajectory (Cui et al., 2019). The severe suppression status of the immune system hampers the patient from clearing the primary infection and increases susceptibility toward secondary and opportunistic infections, thereby leading to many adverse clinical consequences.

Currently, mHLA-DR is a reliable biomarker for evaluating immunosuppression and is widely utilized to guide immunomodulation therapies. Unfortunately, innumerable clinical trials of promising immunostimulation therapies have failed to achieve the desired effect and the consensus is that heterogeneity, especially in individual immune statuses, is responsible for these dismal failures. Due to single biomarkers with limited statistical power, multiple molecular signatures appear to provide better predictive information. Surprisingly, the HLA classifier is closely associated with the immunesuppressive state from multiple perspectives, including infiltrating immune cells, immune-related pathways, and cytokines level, which may act as an effective indicator of immunological paralysis.

One hallmark is apoptosis of B cells and dendritic cells and the depletion and exhaustion of T lymphocytes during sepsis-induced immunoparalysis resulting in an acquired immune deficiency syndrome that is associated with poor outcomes (Hotchkiss et al., 2013). Similarly, deficiency of T helper cells (Th1, Th2, and Th17 cells) proves detrimental to sepsis patients by promoting immunoparalysis, which is associated with increased mortality (Wu et al., 2013). Analogously, the reduced NK cell number and dysfunction may impair the host’s defense against pathogens and make them more vulnerable to nosocomial infection, which participates in sepsis-induced immunosuppression (Kjaergaard et al., 2015). In our study, B cells, activated dendritic cells, ILs, T helper cells, and NK cells were more abundant in HLA low-risk phenotypes than in HLA high-risk phenotypes, and were significantly negatively correlated with the HLA classifier, which is in accordance with the feature of immunosuppression. Conversely, Treg cells that are upregulated in the immunoparalysis stage of sepsis, maintain self-tolerance via inhibiting/suppressing neutrophils, monocytes, and effector T cells, which are associated with clinical worsening and mortality (Kumar, 2018). Likewise, MDSCs, a heterogeneous population of inducible immature myeloid cells with immunosuppressive properties (such as inducing the expansion of Treg cells and suppressing T-cell responses), are expanded during sepsis and serve as one of the contributing factors for sepsis-associated mortality (Schrijver et al., 2019). In our research, Treg cells and MDSCs were significantly more enriched in HLA high-risk endotypes than in HLA low-risk endotypes, and were significantly positively correlated with the HLA classifier, which is in accordance with the characteristic of immunoparalysis. In total, the HLA classifier is negatively associated with activated immune cells defending against infectious, while is positively associated with immunosuppression cells.

Intriguingly, from multiple transcriptome profiles, all of the different immune-related gene sets were significantly enriched in HLA low-risk phenotypes, such as CCR, cytolytic activity, HLA, inflammation-promoting, parainflammation, MHC class I, antigen processing machinery, and APC costimulation, particularly CCR, inflammation-promoting, APC costimulation, and HLA. That is to say, HLA high-risk endotypes were characterized by immunosuppression where numerous activated immune pathways were inhibited compared to HLA low-risk endotypes. In addition, the HLA classifier was significantly negatively associated with CCR, inflammation-promoting, APC costimulation, and HLA, which hints that the HLA classifier can serve as a surrogate marker of sepsis-induced immunosuppression.

Cytokines are one of the key causes underlying sepsis-related immunosuppression and produced by immune cells. During sepsis, a maladjusted and excessive release of pro-inflammatory and anti-inflammatory cytokines will result in a cytokine storm in the early stage of sepsis. However, in the immunosuppression stage of sepsis, the release of proinflammatory cytokines is usually reduced, yet the release of anti-inflammatory cytokines is increased or unchanged, which is generally considered as “immunoparalysis” (or endotoxin tolerance). In our study, pro-inflammatory cytokines (IFNG, IL1B, and TNF) were upregulated in the HLA low-risk subgroup, whereas anti-inflammatory cytokines (IL-10) were upregulated in the HLA high-risk subgroup, which is in keeping with the feature of immunocompromise. Additionally, elevated ratios of anti-inflammatory and pro-inflammatory cytokines (e.g., IL-10/TNF) are proposed markers of sepsis-induced immunosuppression and are associated with multiple organ failure (Loisa et al., 2003). Notably, the HLA classifier was significantly positively related to ratios of IL-10/TNF in our study, which implies that the HLA classifier can act as a promising biomarker of sepsis-induced immunoparalysis.

Taken together, according to immune cell infiltration, immune-related pathways, and cytokines level, the HLA classifier could efficiently reflect immunological status, which may help guide immune-modulating agents to achieve immune homeostasis.

In spite of the remarkable results, it is inevitable that limitations also existed in our research. First, though our model, based on multiple transcriptome data, demonstrated impressive performance in early diagnosis, identification of high-risk patients, and recognition of immunosuppression for sepsis, it is not yet suitable for general use prior to validation of external datasets with large sample sizes in prospective cohorts. Second, patients with sepsis included in our analysis were not guaranteed to be free of other diseases. Whereas, the influence of other diseases on our results cannot be fully resolved because the original data set did not offer complete details of other comorbidities/diseases. Third, based on bulk RNA-seq data, the CIBERSORTx deconvolution algorithm and ssGSEA with metagenes may not accurately identify immune cell subpopulations although different methods and different data sets validate each other. It is necessary to use flow cytometry or single-cell RNA-seq methods or fluorescence-activated cell sorting to verify our results. Fourth, no further *in vivo* experiments were conducted to validate these results (hub HLA genes, immune infiltration cells, and pivotal molecular pathways). Loss of function and overexpression studies *in vitro*, as well as in animal models, will help to further identify the exact role of hub HLA genes in the regulation of the inflammatory response and related pathogenic signaling in sepsis.

## Conclusion

A diagnostic and prognostic model, namely the HLA classifier, was established based on five HLA genes that were closely correlated with responses to hydrocortisone and immunosuppression state, and might facilitate individualized interventions for specific therapy.

## Data Availability

The original contributions presented in the study are included in the article/Supplementary Material, further inquiries can be directed to the corresponding authors.

## Supplementary Material

Supplementary Figure S1 Flowchart of dataset selection.

Supplementary Figure S2 Hub HLA genes selected by Lasso regression analysis and random forest (RF) in GSE63042 datasets. **(A)** The two dotted vertical lines are drawn at the optimal values by minimum criteria (left) and 1—s.e. criteria (right). **(B)** Lasso coefficient profiles of the seven hub HLA genes. A vertical line is drawn at the optimal value by minimum criteria and results in seven non-zero coefficients. **(C)** Distribution diagram of regression tree and error. **(D)** Important variables ranked by IncNodePurity.

Supplementary Figure S3 Hub HLA genes selected by Lasso regression analysis and random forest (RF) in GSE95233 datasets. **(A)** The two dotted vertical lines are drawn at the optimal values by minimum criteria (left) and 1—s.e. criteria (right). **(B)** Lasso coefficient profiles of the 15 hub HLA genes. A vertical line is drawn at the optimal value by minimum criteria and results in 15 non-zero coefficients. **(C)** Distribution diagram of regression tree and error. **(D)** Important variables ranked by IncNodePurity.

Supplementary Figure S4 The location of 5 hub HLA genes on 23 chromosomes.

Supplementary Figure S5 The distribution of mortality rate in different HLA subgroups and the prognostic capacity of the HLA classifier in external validation cohorts. **(A,B)** E-MTAB-4421 datasets. **(C,D)** E-MTAB-4451 datasets. **(E,F)**: E-MTAB-7581 datasets.

Supplementary Figure S6 The evaluation of the clinical usefulness of the HLA classifier in the discovery cohorts. **(A)** GSE54514 datasets. **(B)** GSE63042 datasets.

Supplementary Figure S7 The distribution of 22 immune infiltration cells in each sepsis sample from GSE65682 datasets.

Supplementary Figure S8 The distribution of 22 immune infiltration cells in each sepsis sample from E-MTAB-4421 datasets.

Supplementary Figure S9 The distribution of 22 immune infiltration cells in each sepsis sample from E-MTAB-4451 datasets.

Supplementary Figure S10 Bar plot displays differences in pathway activities between different HLA subgroups.

## Abbreviations

APACHE II, acute physiology and chronic health evaluation; APC, antigen-presenting cell; CCR, cytokine-cytokine receptor interaction; CRP, C-reactive protein; DCA, decision curve analysis; DM, diabetes mellitus; GEO, gene expression omnibus; GSVA, gene set variation analysis; HLA, human leukocyte antigen; IL, infiltrating lymphocyte; MARS, molecular diagnosis and risk stratification of sepsis; MDSCs, myeloid-derived suppressor cells; mHLA-DR, monocyte HLA-DR; MSigDB, molecular signatures Database; PCT, procalcitonin; qSOFA, quick sequential organ failure; RF, random forest; ROC, receiver operating characteristic; SSC, surviving sepsis campaign; ssGSEA, single-sample gene set enrichment analysis; SRS, sepsis response signature.
