# Supplementary figures and images for: Construction of an HLA Classifier for Early Diagnosis, Prognosis, and Recognition of Immunosuppression in Sepsis by Multiple Transcriptome Datasets
Source: Front Physiol. 2022 May 24;13:870657. doi: 10.3389/fphys.2022.870657 (PMC9171028; doi:10.3389/fphys.2022.870657)

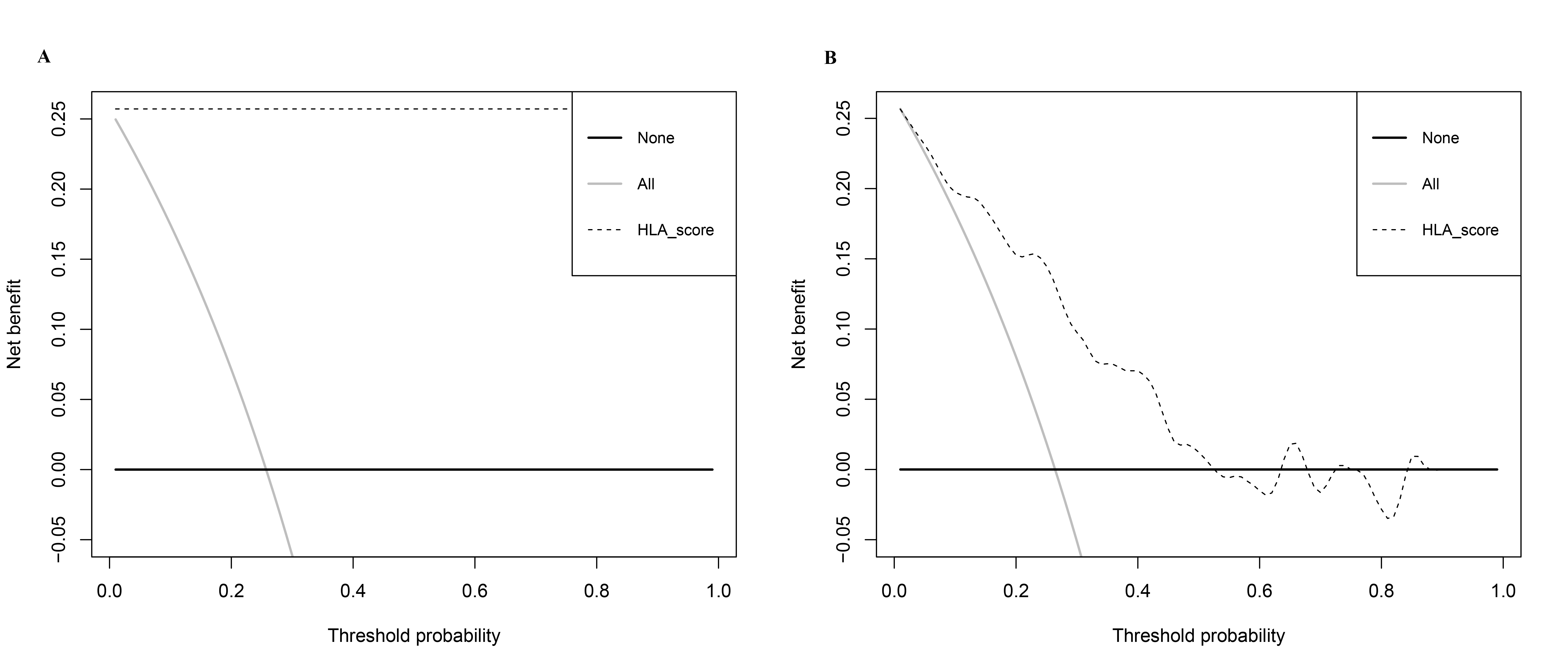

Supplement: Supplementary file 3 [file Image6.TIF]

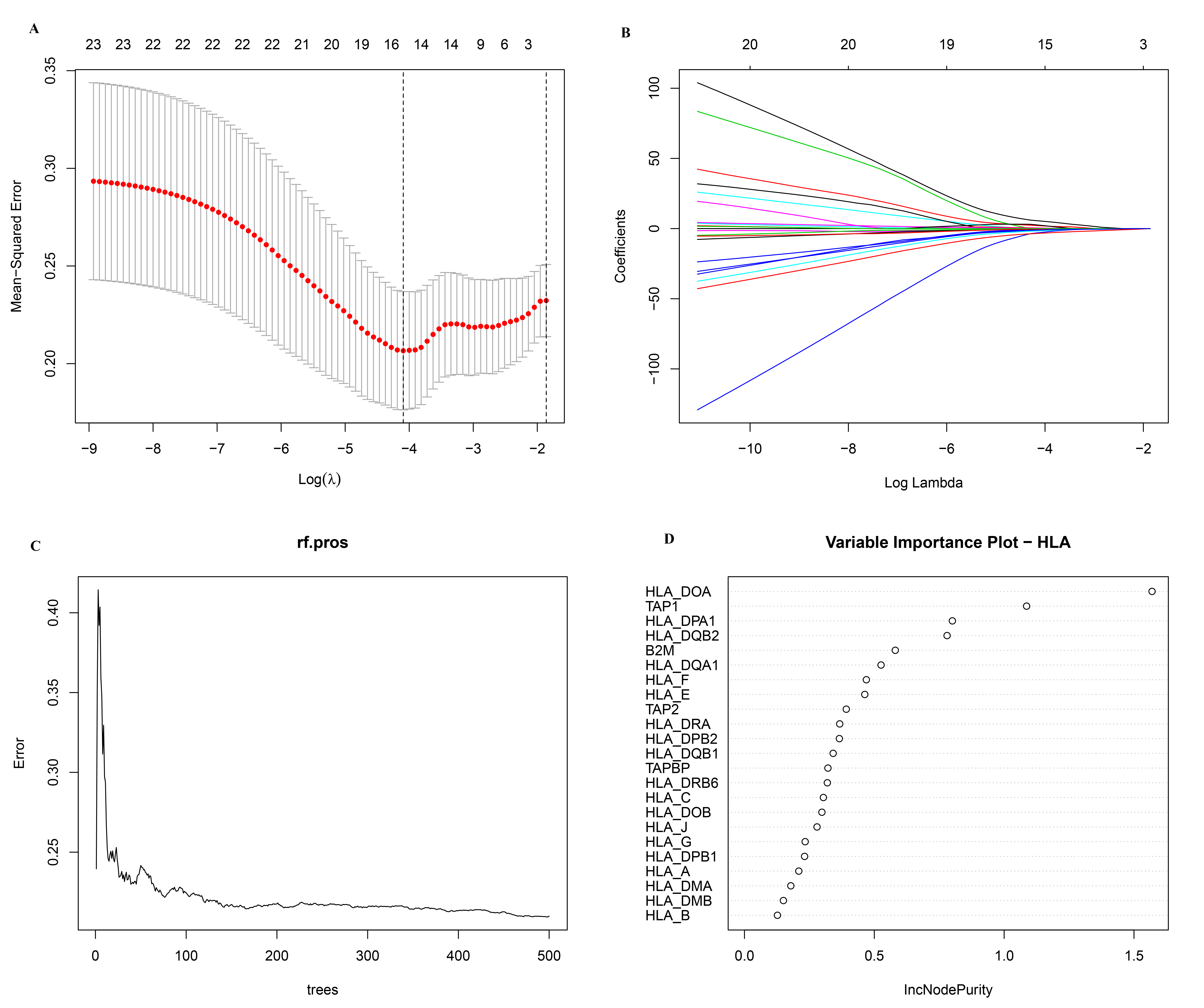

Supplement: Supplementary file 4 [file Image3.TIF]

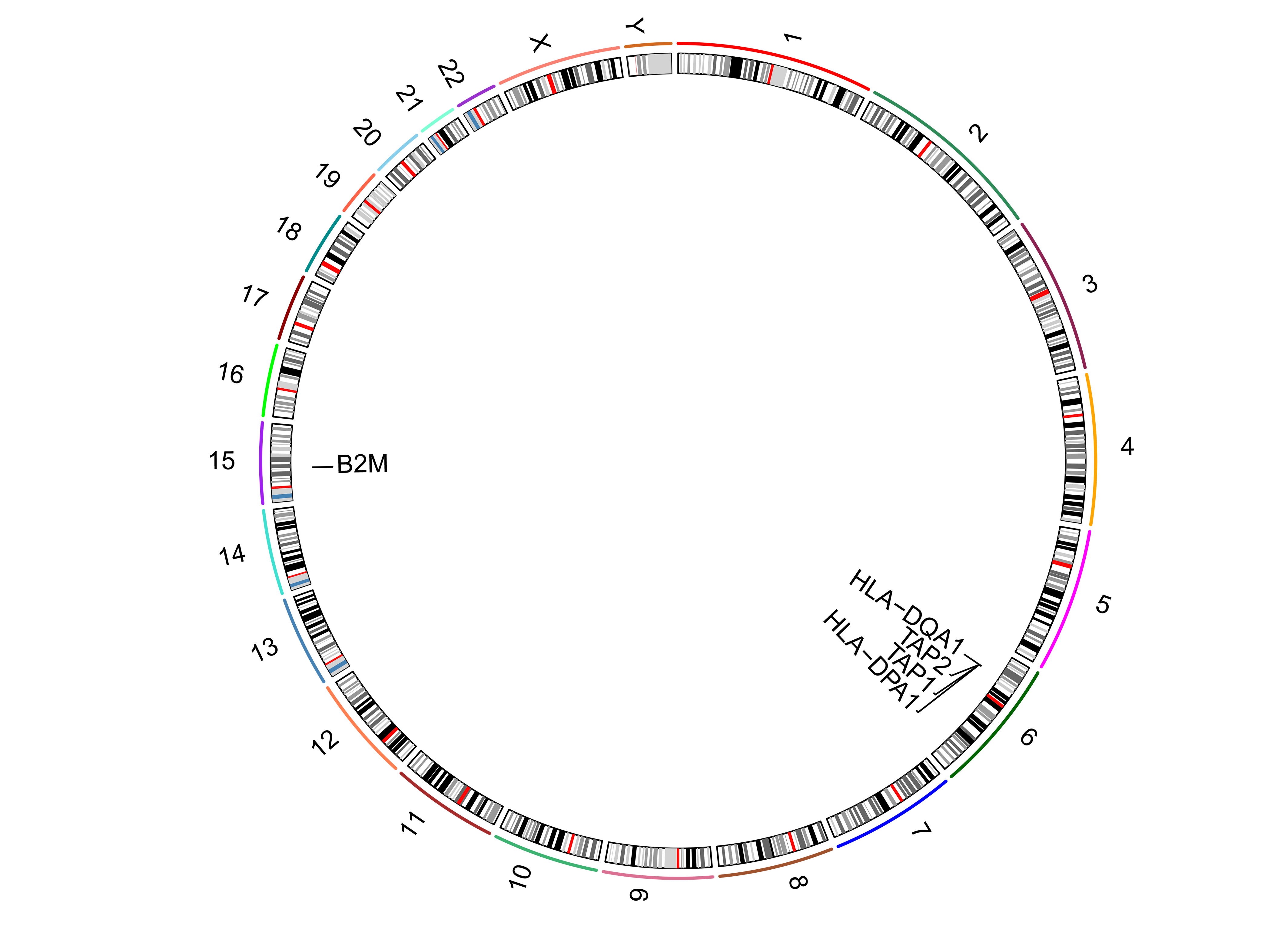

Supplement: Supplementary file 5 [file Image4.TIF]

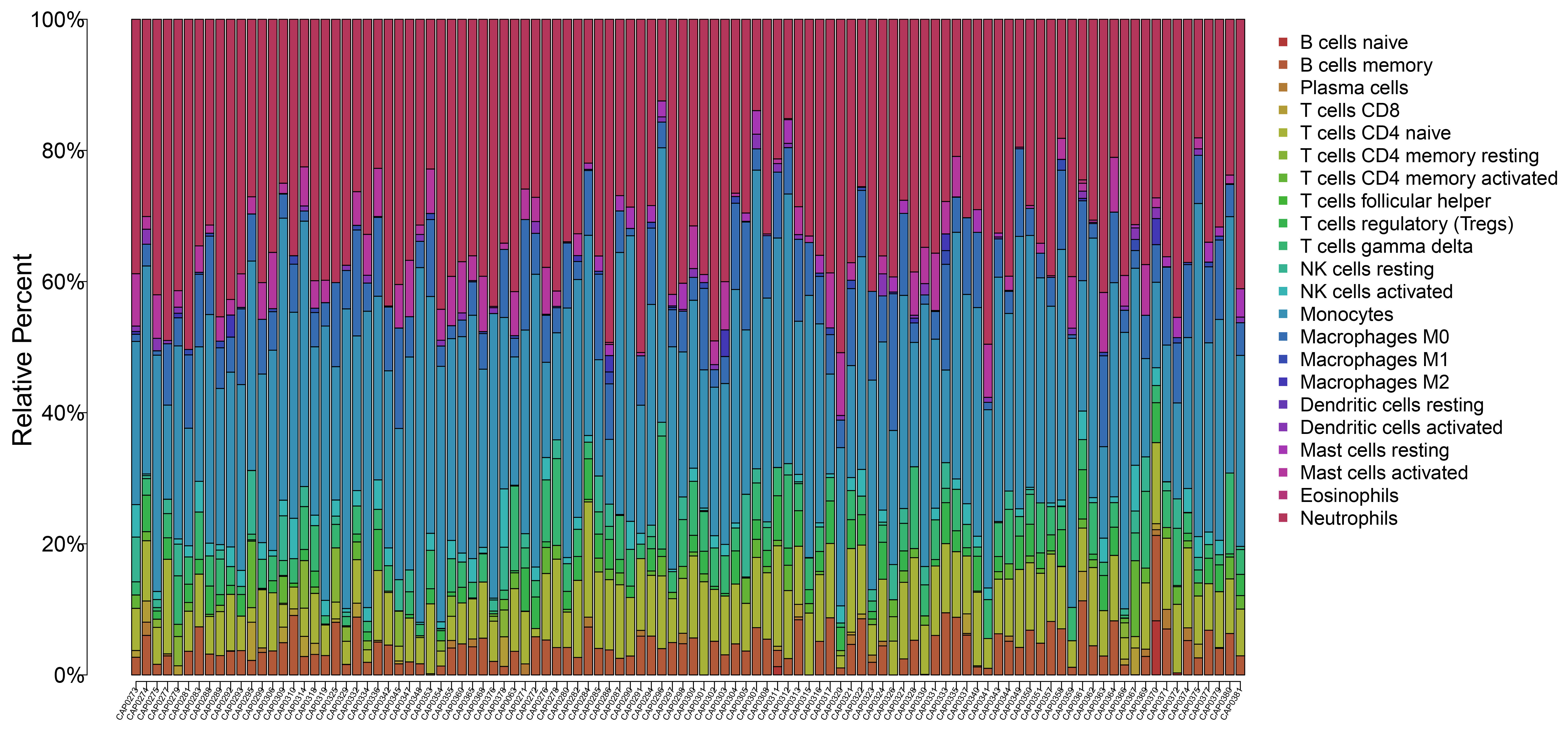

Supplement: Supplementary file 6 [file Image9.TIF]

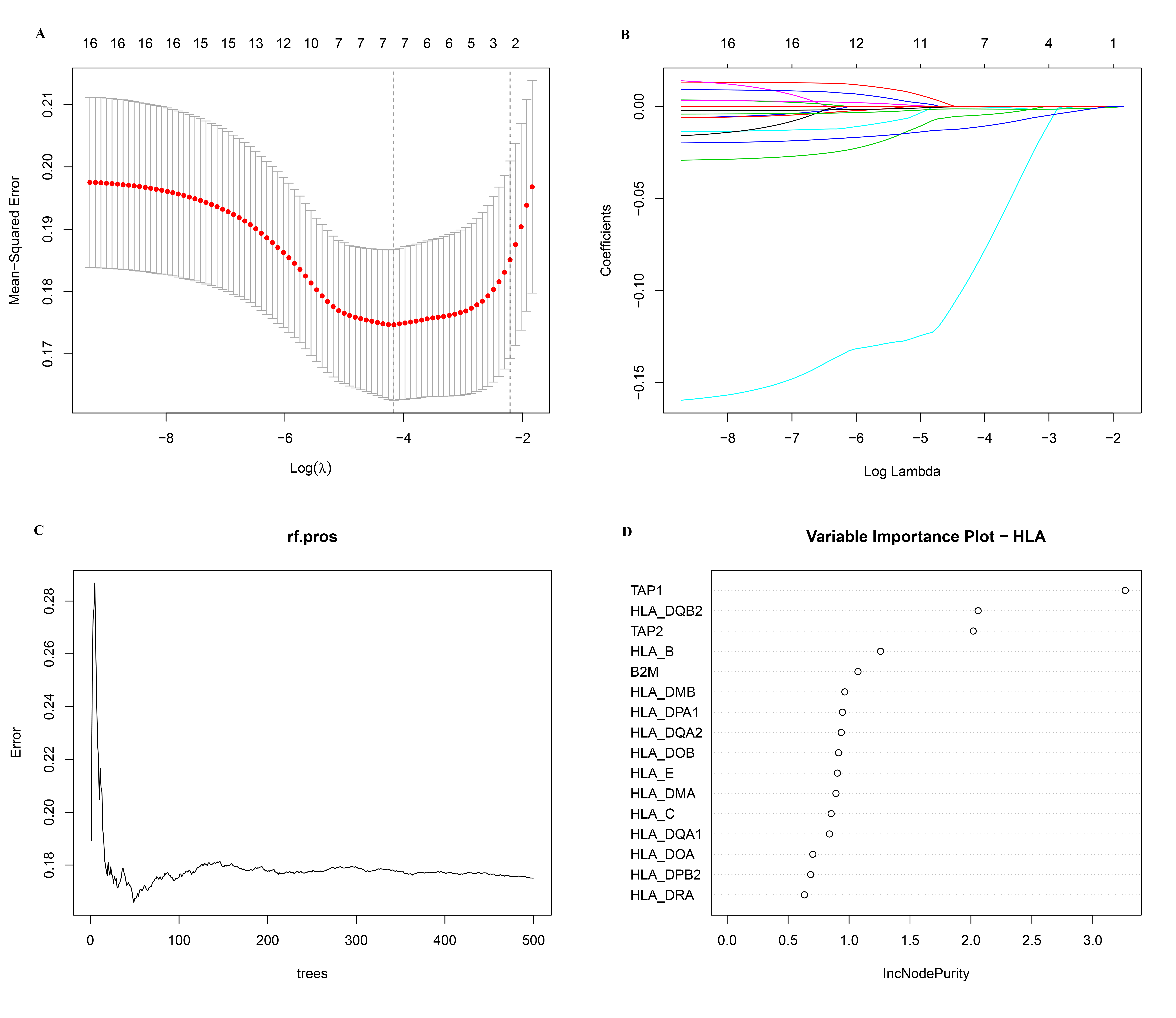

Supplement: Supplementary file 7 [file Image2.TIF]

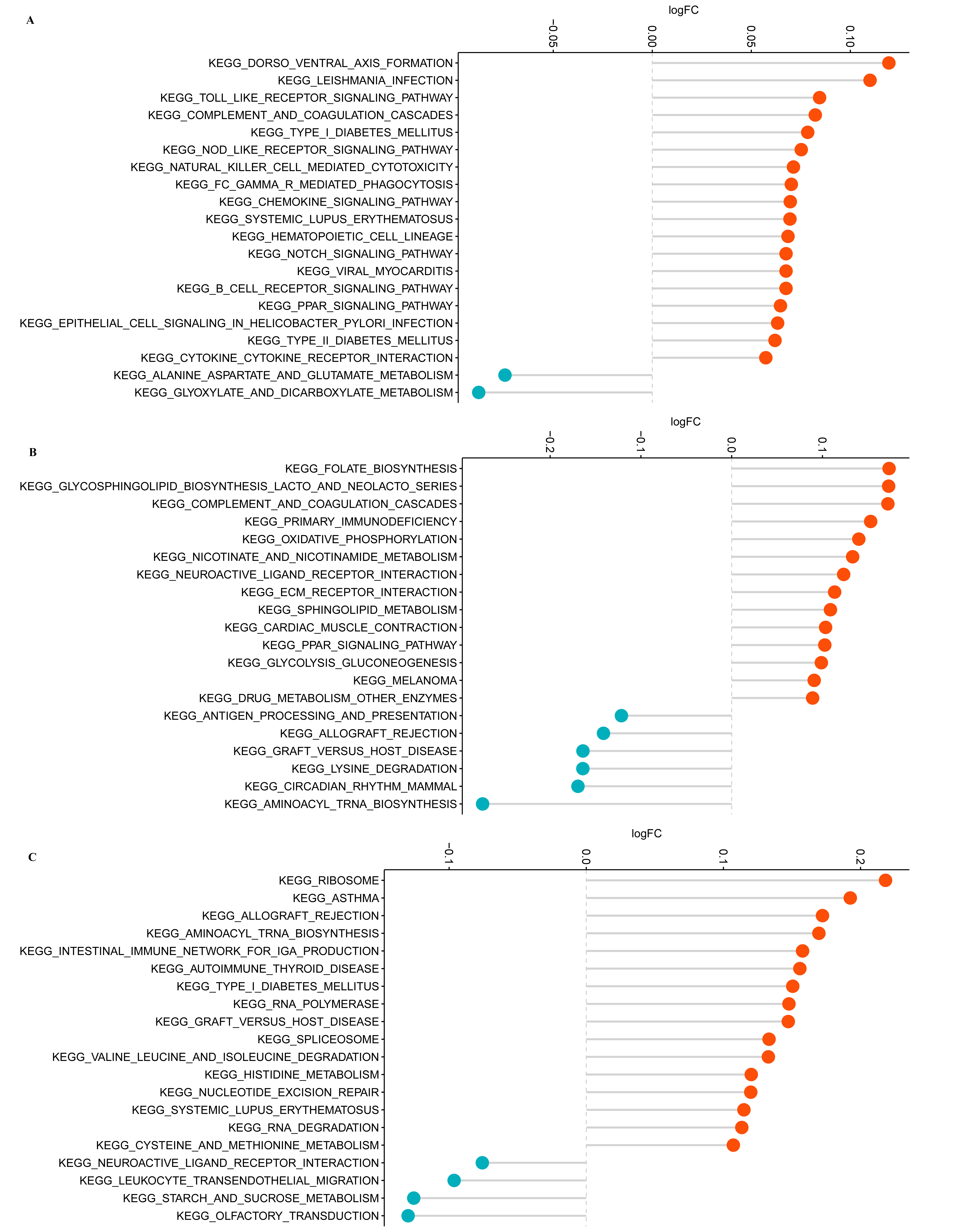

Supplement: Supplementary file 10 [file Image10.TIF]

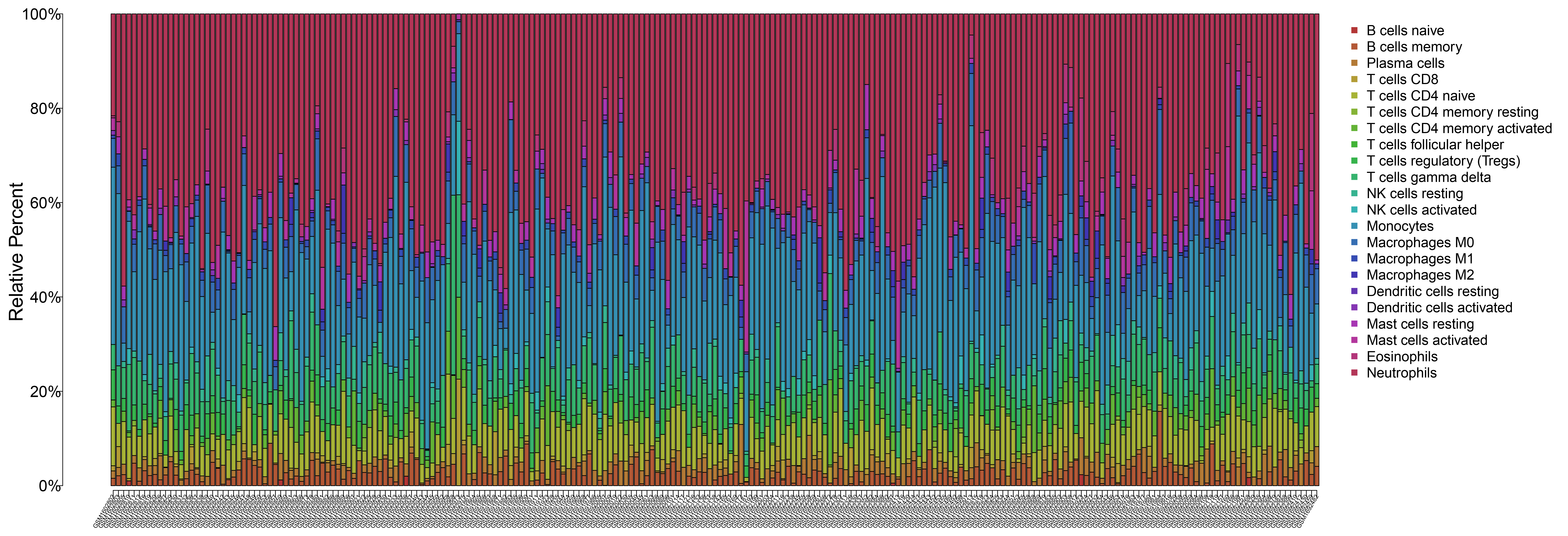

Supplement: Supplementary file 11 [file Image7.TIF]

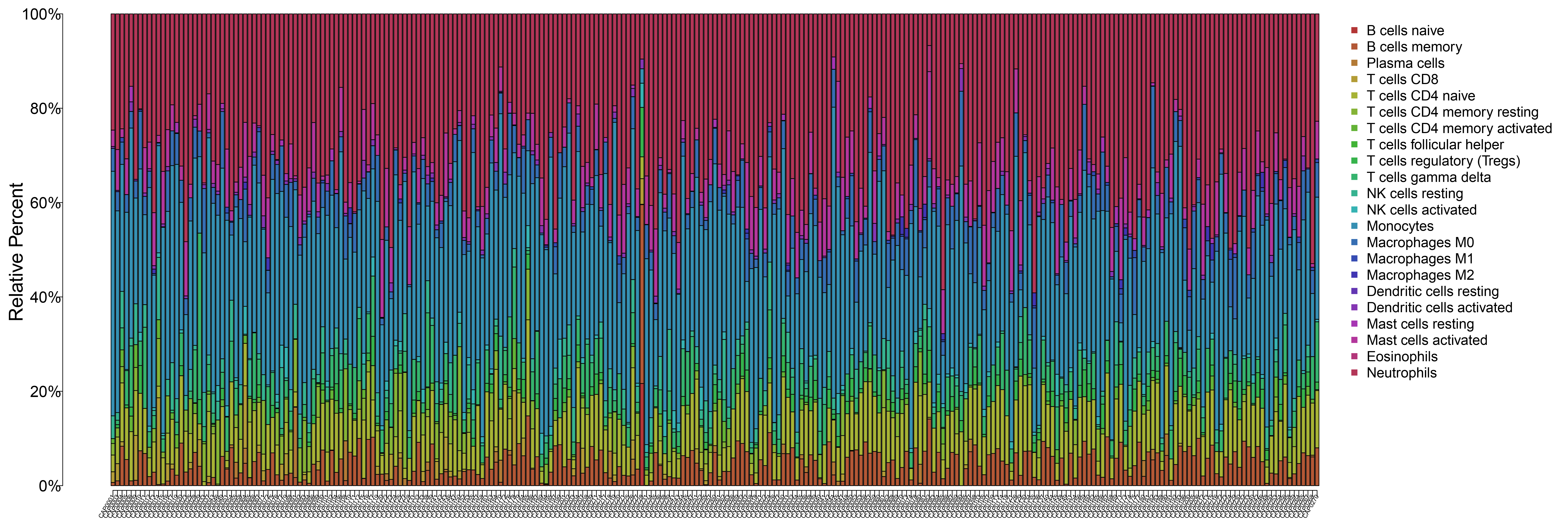

Supplement: Supplementary file 14 [file Image8.TIF]

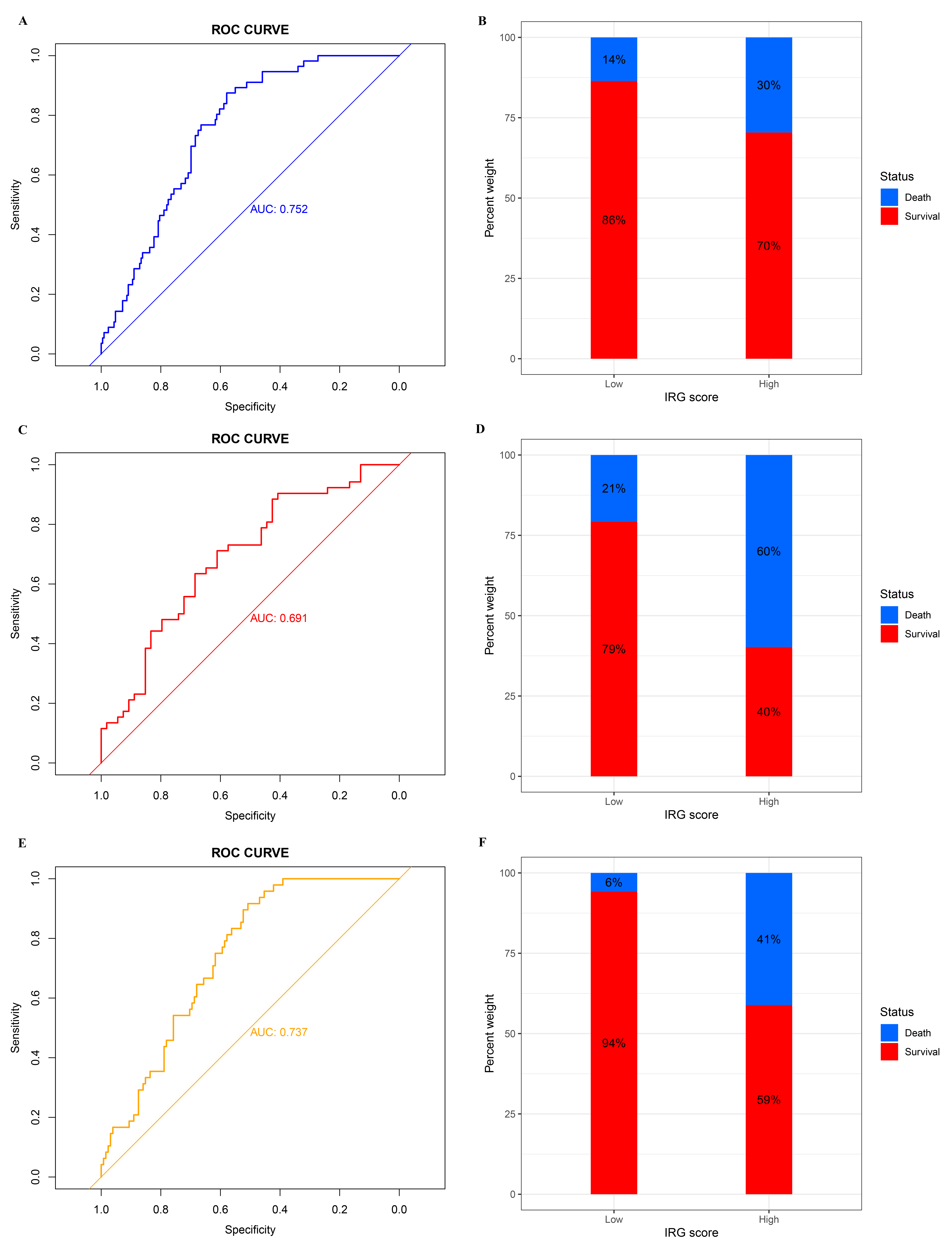

Supplement: Supplementary file 15 [file Image5.TIF]
