# Supplementary material for: Construction of an HLA Classifier for Early Diagnosis, Prognosis, and Recognition of Immunosuppression in Sepsis by Multiple Transcriptome Datasets
Source: Front Physiol. 2022 May 24;13:870657. doi: 10.3389/fphys.2022.870657 (PMC9171028; doi:10.3389/fphys.2022.870657)
Supplement: Supplementary file 16 [file DataSheet2.doc]

Supplementary Material 5

**数据合并**

setwd("F:\\科研论文\\科研学术\\ICU生信研究\\Sepsis\\HLA\\数据分析\\差异分析\\GSE65682")

inputfile1="HLA.txt" #生存时间数据

inputfile2="geneMatrix.txt" #差异基因表达数据

time_data<-read.table(inputfile1,header = T,sep = "\t",check.names = F)

geneEXP<-read.table(inputfile2,header = T,sep = "\t",check.names = F)

head(time_data)

head(geneEXP)

merger_data<-merge(time_data,geneEXP,by="id")

write.table(merger_data,"HLAExp.txt",sep = "\t",row.names = F,quote = F)

**差异分析**

setwd("F:\\科研论文\\科研学术\\ICU生信研究\\Sepsis\\HLA\\数据分析\\差异分析\\GSE65682")

#设置工作目录

library(vioplot) #引用包

library(limma)

rt=read.table("HLAEx.txt",sep="\t",header=T,row.names=1,check.names=F) #读取输入文件

normal=42 #正常样品数目

tumor=242 #肿瘤样品数目

pdf("HLA.pdf",height=8,width=14) #保存图片的文件名称

par(las=1,mar=c(10,6,3,3))

x=c(1:ncol(rt))

y=c(1:ncol(rt))

plot(x,y,

xlim=c(0,58),ylim=c(min(rt),max(rt)+0.02),

main="",xlab="", ylab="Expression",

pch=21,

col="white",

xaxt="n")

#对每个免疫细胞循环，绘制vioplot，正常用绿色表示，肿瘤用红色表示

for(i in 1:ncol(rt)){

normalData=rt[1:normal,i]

tumorData=rt[(normal+1):(normal+tumor),i]

vioplot(normalData,at=3*(i-1),lty=1,add = T,col = 'blue')

vioplot(tumorData,at=3*(i-1)+1,lty=1,add = T,col = 'red')

wilcoxTest=wilcox.test(normalData,tumorData)

p=round(wilcoxTest$p.value,3)

mx=max(c(normalData,tumorData))

lines(c(x=3*(i-1)+0.2,x=3*(i-1)+0.8),c(mx,mx))

text(x=3*(i-1)+0.5,y=mx+0.15,labels=ifelse(p<0.001,paste0("p<0.001"),paste0("p=",p)),cex = 0.8)

text(seq(1,58,3),0.6,xpd = NA,labels=colnames(rt),cex = 1,srt = 45,pos=2)

}

dev.off()

**RCircos**

setwd("F:\\科研论文\\科研学术\\ICU生信研究\\Sepsis\\HLA\\数据分析\\RCircos") #设置工作目录

inputfile1="GeneRef.txt" #生存时间数据

inputfile2="intersectGene.txt" #差异基因表达数据

time_data<-read.table(inputfile1,header = T,sep = "\t",check.names = F)

geneEXP<-read.table(inputfile2,header = T,sep = "\t",check.names = F)

head(time_data)

head(geneEXP)

merger_data<-merge(time_data,geneEXP,by="Gene")

write.table(merger_data,"GeneLabel.txt",sep = "\t",row.names = F,quote = F)

setwd("F:\\科研论文\\科研学术\\ICU生信研究\\Sepsis\\HLA\\数据分析\\RCircos")

library("RCircos") #引用包

#初始化圈图

#Initialize RCircos core components

cytoBandIdeogram=read.table("Refer.txt",sep="\t",header=T)

chr.exclude <- NULL

cyto.info <- cytoBandIdeogram

tracks.inside <- 10

tracks.outside <- 0

RCircos.Set.Core.Components(cyto.info, chr.exclude, tracks.inside, tracks.outside)

RCircos.List.Plot.Parameters()

#设置圈图参数

rcircos.params <- RCircos.Get.Plot.Parameters()

rcircos.params$text.size=1

rcircos.params$point.size=5

#rcircos.params$heatmap.color <- "GreenWhiteRed"

RCircos.Reset.Plot.Parameters(rcircos.params)

RCircos.List.Plot.Parameters()

pdf(file="RCircos.pdf", height=8, width=8)

RCircos.Set.Plot.Area()

RCircos.Chromosome.Ideogram.Plot()

#Gene Labels

RCircos.Gene.Label.Data=read.table("GeneLabel.txt",sep="\t",header=T)

name.col <- 4

side <- "in"

track.num <- 1.5

RCircos.Gene.Connector.Plot(RCircos.Gene.Label.Data,track.num, side)

track.num <- 2.5

RCircos.Gene.Name.Plot(RCircos.Gene.Label.Data,name.col,track.num, side)

dev.off()

**Lasso回归-Logistic Regression**

setwd("F:\\科研论文\\科研学术\\ICU生信研究\\Sepsis\\HLA\\数据分析\\特征选择\\LASSO\\GSE65682")

#设置工作目录

inputfile1="Clinical.txt" #生存时间数据

inputfile2="HLAEx.txt" #差异基因表达数据

time_data<-read.table(inputfile1,header = T,sep = "\t",check.names = F)

geneEXP<-read.table(inputfile2,header = T,sep = "\t",check.names = F)

head(time_data)

head(geneEXP)

merger_data<-merge(time_data,geneEXP,by="id")

write.table(merger_data,"HLAE.txt",sep = "\t",row.names = F,quote = F)

library(glmnet)

setwd("F:\\科研论文\\科研学术\\ICU生信研究\\Sepsis\\HLA\\数据分析\\特征选择\\LASSO\\GSE65682")

lncRNA<-read.table("HLAE.txt",header=T,sep="\t",row.names = 1,check.names = F,stringsAsFactors = F)

#lncRNAEXP=log2(lncRNA[,2:ncol(lncRNA)]+1)

#lncRNA=cbind(lncRNA[1],lncRNAEXP)

v1<-as.matrix(lncRNA[,c(2:ncol(lncRNA))])

v2 <- as.matrix(lncRNA$Status)

myfit <- glmnet(v1, v2, alpha=1,family='binomial')

plot(myfit, xvar = "lambda", label =FALSE)

myfit1 <- cv.glmnet(v1, v2, alpha=1)

plot(myfit1)

abline(v=log(c(myfit1$lambda.min,myfit1$lambda.1se)),lty="dashed")

myfit1$lambda.min

coe <- coef(myfit, s = myfit1$lambda.min)

act_index <- which(coe != 0)

act_coe <- coe[act_index]

row.names(coe)[act_index]

myfit1$lambda.min

myfit1$lambda.1se

coef(myfit1,s="lambda.min")

**RF**

library(randomForest) #random forests

library(caret) #

setwd("F:\\科研论文\\科研学术\\ICU生信研究\\Sepsis\\HLA\\数据分析\\特征选择\\RF\\GSE65682")

data=read.table("HLAE.txt",sep="\t",header=T,check.names=F, row.names = 1)

rf.pros <- randomForest(Status ~ ., data = data)

rf.pros

plot(rf.pros)

which.min(rf.pros$mse)

rf.pros.2 <- randomForest(Status~ ., data =data, ntree = 230)

rf.pros.2

varImpPlot(rf.pros.2, scale = TRUE,

main = "Variable Importance Plot - HLA")

importance(rf.pros.2)

**UpSetR**

setwd("F:\\科研论文\\科研学术\\ICU生信研究\\Sepsis\\HLA\\数据分析\\特征选择\\UpSetR")

#设置工作目录

library(UpSetR) #加载包

outFile="intersectGenes.txt" #输出交集基因文件

files=dir() #获取目录下所有文件

files=grep("txt$",files,value=T) #提取.txt结尾的文件

geneList=list()

#获取所有txt文件中的基因信息，保存到geneList

for(i in 1:length(files)){

inputFile=files[i]

if(inputFile==outFile){next}

rt=read.table(inputFile,header=F) #读取输入文件

geneNames=as.vector(rt[,1]) #提取基因名称

geneNames=gsub("^ | $","",geneNames) #去掉基因首尾的空格

uniqGene=unique(geneNames) #基因取unique，唯一基因列表

header=unlist(strsplit(inputFile,"\\.|\\-"))

geneList[[header[1]]]=uniqGene

uniqLength=length(uniqGene)

print(paste(header[1],uniqLength,sep=" "))

}

####第二步、取交集作图#####

upsetData=fromList(geneList)

upset(upsetData,

nsets = length(geneList), #展示多少个数据.

nintersects =50, #展示基因集数目，医学学霸帮为了变成万能代码，就改成这样。

order.by = "freq", #按照数目排序,freq降序，degree升序.

show.numbers = "yes", #柱状图上方是否显示数值

number.angles = 0, #字体角度

point.size = 2, #点的大小

matrix.color="red", #交集点颜色

line.size = 0.8, #线条粗细

mainbar.y.label = "Gene Intersections",

sets.x.label = "Set Size",

text.scale = c(1.5, 1.5, 1.5, 1.5, 1.5, 1.5))

####第三步、输出交集结果#####

intersectGenes=Reduce(intersect,geneList)

write.table(file=outFile,intersectGenes,sep="\t",quote=F,col.names=F,row.names=F)

**dROC**

library(rms)

library(foreign)

setwd("F:\\科研论文\\科研学术\\ICU生信研究\\Sepsis\\HLA\\数据分析\\dROC\\GSE57065")

data=read.table("dROC.txt",sep="\t",header=T,check.names=F,row.names = 1)

ddist <- datadist(data)

options(datadist='ddist')

modelA <- glm(Status~., data = data, family = binomial(link="logit"))

summary(modelA)

cbind(coef= coef(modelA),confint(modelA))

exp(cbind(OR= coef(modelA),confint(modelA)))

Score<- predict(newdata=data,modelA,"response")

write.table(cbind(id=rownames(cbind(data[,1:6],Score)),cbind(data[,1:6],Score)),"RiskScore.txt",sep="\t",quote=F,row.names=F)

setwd("F:\\科研论文\\科研学术\\ICU生信研究\\Sepsis\\HLA\\数据分析\\dROC\\GSE57065")

data=read.table("RiskScore.txt",sep="\t",header=T,check.names=F,row.names = 1)

library(pROC)

gmodelA <- roc(Status~Score, data = data,smooth=F)

plot(gmodelA, print.auc=TRUE, print.thres=TRUE,main = "ROC CURVE", col= "blue",print.thres.col="blue",identity.col="blue",

identity.lty=1,identity.lwd=1)

plot(gmodelA, print.auc=TRUE, main = "ROC CURVE", col= "blue",print.thres.col="blue",identity.col="blue",

identity.lty=1,identity.lwd=1)

**pROC曲线**

setwd("F:\\科研论文\\科研学术\\ICU生信研究\\Sepsis\\HLA\\数据分析\\pROC\\GSE54514")

inputfile1="HLA.txt" #生存时间数据

inputfile2="geneMatrix.txt" #差异基因表达数据

time_data<-read.table(inputfile1,header = T,sep = "\t",check.names = F)

geneEXP<-read.table(inputfile2,header = T,sep = "\t",check.names = F)

head(time_data)

head(geneEXP)

merger_data<-merge(time_data,geneEXP,by="id")

write.table(merger_data,"HLAExp.txt",sep = "\t",row.names = F,quote = F)

library(rms)

library(foreign)

setwd("F:\\科研论文\\科研学术\\ICU生信研究\\Sepsis\\HLA\\数据分析\\pROC\\GSE54514")

data=read.table("pROC.txt",sep="\t",header=T,check.names=F,row.names = 1)

ddist <- datadist(data)

options(datadist='ddist')

modelA <- glm(Status~., data = data, family = binomial(link="logit"))

summary(modelA)

cbind(coef= coef(modelA),confint(modelA))

exp(cbind(OR= coef(modelA),confint(modelA)))

Score<- predict(newdata=data,modelA,"response")

write.table(cbind(id=rownames(cbind(data[,1:6],Score)),cbind(data[,1:6],Score)),"RiskScore.txt",sep="\t",quote=F,row.names=F)

**Bar**

library(plyr)

library(ggplot2)

library(ggpubr)

scoreFile="Bar.txt" #m6A打分文件

trait="Status" #临床性状

setwd("F:\\科研论文\\科研学术\\ICU生信研究\\Sepsis\\HLA\\数据分析\\pROC\\GES63042")

#读取输入文件

rt=read.table(scoreFile, header=T, sep="\t", check.names=F, row.names=1)

#定义临床性状的颜色

bioCol=c("#0066FF","#FF0000","#FF9900","#6E568C","#7CC767","#223D6C","#D20A13","#FFD121","#088247","#11AA4D")

bioCol=bioCol[1:length(unique(rt[,trait]))]

#统计高低评分组病人数目

rt1=rt[,c(trait, "group")]

colnames(rt1)=c("trait", "group")

df=as.data.frame(table(rt1))

#计算高低评分组的百分率

df=ddply(df, .(group), transform, percent = Freq/sum(Freq) * 100)

#百分比位置

df=ddply(df, .(group), transform, pos = (cumsum(Freq) - 0.5 * Freq))

df$label=paste0(sprintf("%.0f", df$percent), "%")

df$group=factor(df$group, levels=c("Low", "High"))

#绘制百分率图

p=ggplot(df, aes(x = factor(group), y = percent, fill = trait)) +

geom_bar(position = position_stack(), stat = "identity", width = .3) +

scale_fill_manual(values=bioCol)+

xlab("IRG score")+ ylab("Percent weight")+ guides(fill=guide_legend(title=trait))+

geom_text(aes(label = label), position = position_stack(vjust = 0.5), size = 4) +

#coord_flip()+

theme_bw()

pdf(file="Bar.pdf", width=6, height=5)

print(p)

dev.off()

**ArrayExpress**

setwd("F:\\科研论文\\科研学术\\ICU生信研究\\Sepsis\\HLA\\数据分析\\ArrayExpress\\E-MTAB-4421")

#设置工作目录

inputfile1="intersectGene.txt" #生存时间数据

inputfile2="geneMatrix.txt" #差异基因表达数据

time_data<-read.table(inputfile1,header = T,sep = "\t",check.names = F)

geneEXP<-read.table(inputfile2,header = T,sep = "\t",check.names = F)

head(time_data)

head(geneEXP)

merger_data<-merge(time_data,geneEXP,by="id")

write.table(merger_data,"ROC.txt",sep = "\t",row.names = F,quote = F)

setwd("F:\\科研论文\\科研学术\\ICU生信研究\\Sepsis\\HLA\\数据分析\\ArrayExpress\\E-MTAB-4421")

#设置工作目录

inputfile1="Clinical.txt" #生存时间数据

inputfile2="ROC.txt" #差异基因表达数据

time_data<-read.table(inputfile1,header = T,sep = "\t",check.names = F)

geneEXP<-read.table(inputfile2,header = T,sep = "\t",check.names = F)

head(time_data)

head(geneEXP)

merger_data<-merge(time_data,geneEXP,by="id")

write.table(merger_data,"pROC.txt",sep = "\t",row.names = F,quote = F)

library(rms)

library(foreign)

setwd("F:\\科研论文\\科研学术\\ICU生信研究\\Sepsis\\HLA\\数据分析\\ArrayExpress\\E-MTAB-4421")

data=read.table("ROC.txt",sep="\t",header=T,check.names=F,row.names = 1)

#dataEXP=log2(data[,2:ncol(data)]+1)

#data=cbind(data[1],dataEXP)

ddist <- datadist(data)

options(datadist='ddist')

modelA <- glm(Status~., data = data, family = binomial(link="logit"))

summary(modelA)

cbind(coef= coef(modelA),confint(modelA))

exp(cbind(OR= coef(modelA),confint(modelA)))

Score<- predict(newdata=data,modelA,"response")

write.table(cbind(id=rownames(cbind(data[,1:6],Score)),cbind(data[,1:6],Score)),"RiskScore.txt",sep="\t",quote=F,row.names=F)

data=read.table("RiskScore.txt",sep="\t",header=T,check.names=F,row.names = 1)

library(pROC)

gmodelA <- roc(Status~Score, data = data,smooth=F)

plot(gmodelA, print.auc=TRUE, print.thres=TRUE,main = "ROC CURVE", col= "blue",print.thres.col="blue",identity.col="blue",

identity.lty=1,identity.lwd=1)

plot(gmodelA, print.auc=TRUE, main = "ROC CURVE", col= "blue",print.thres.col="blue",identity.col="blue",

identity.lty=1,identity.lwd=1)

library(plyr)

library(ggplot2)

library(ggpubr)

scoreFile="Bar.txt" #m6A打分文件

trait="Status" #临床性状

setwd("F:\\科研论文\\科研学术\\ICU生信研究\\Sepsis\\HLA\\数据分析\\ArrayExpress\\E-MTAB-4421")

#读取输入文件

rt=read.table(scoreFile, header=T, sep="\t", check.names=F, row.names=1)

#定义临床性状的颜色

bioCol=c("#0066FF","#FF0000","#FF9900","#6E568C","#7CC767","#223D6C","#D20A13","#FFD121","#088247","#11AA4D")

bioCol=bioCol[1:length(unique(rt[,trait]))]

#统计高低评分组病人数目

rt1=rt[,c(trait, "group")]

colnames(rt1)=c("trait", "group")

df=as.data.frame(table(rt1))

#计算高低评分组的百分率

df=ddply(df, .(group), transform, percent = Freq/sum(Freq) * 100)

#百分比位置

df=ddply(df, .(group), transform, pos = (cumsum(Freq) - 0.5 * Freq))

df$label=paste0(sprintf("%.0f", df$percent), "%")

df$group=factor(df$group, levels=c("Low", "High"))

#绘制百分率图

p=ggplot(df, aes(x = factor(group), y = percent, fill = trait)) +

geom_bar(position = position_stack(), stat = "identity", width = .3) +

scale_fill_manual(values=bioCol)+

xlab("IRG score")+ ylab("Percent weight")+ guides(fill=guide_legend(title=trait))+

geom_text(aes(label = label), position = position_stack(vjust = 0.5), size = 4) +

#coord_flip()+

theme_bw()

pdf(file="Bar.pdf", width=6, height=5)

print(p)

dev.off()

**mROC**

setwd("F:\\科研论文\\科研学术\\ICU生信研究\\Sepsis\\HLA\\数据分析\\mROC\\E-MTAB-4421")

library(pROC) # 加载pROC包
library(ggplot2) # 调用ggplot2包以利用ggroc函数

data=read.table("SPSS.txt",sep="\t",header=T,check.names=F,row.names = 1)

roc.list <- roc(Status ~ Age + SRS + HLA_score, data = data)
ggroc1 <- ggroc(roc.list,
legacy.axes = TRUE);

ggroc1
ggroc2<-ggroc1 + xlab("1-specificity") + ylab("Sensitivity") +theme_bw()+ theme(panel.grid.major=element_line(colour=NA),

panel.grid.minor = element_blank()) + geom_segment(aes(x = 0,xend = 1,y = 0,yend = 1),col = "darkgrey",linetype = "dashed")

ggroc3<-ggroc2+

theme(legend.position=c(0.85,0.20))+#设置图例位置

theme(legend.text=element_text(size=10,face="plain"))+#设置图例字体格式

theme(legend.background=element_rect(fill="white",colour="black"))#图例填充白色，边框为黑色

ggroc3

ggroc4<-ggroc3+scale_y_continuous(expand=c(0,0))+scale_x_continuous(expand=c(0,0))

ggroc4

roc1 <- roc(data$Status,data$Age);roc1 # Build a ROC object and compute the AUC
roc2 <- roc(data$Status,data$SRS);roc2

roc3 <- roc(data$Status,data$HLA_score);roc3

ggroc4+annotate("text",x=0.6,y=0.40,label="AUC=0.390",size=3)+

annotate("text",x=0.6,y=0.60,label="AUC=0.504",size=3)+

annotate("text",x=0.6,y=0.8,label="AUC=0.683",size=3)

setwd("F:\\科研论文\\科研学术\\ICU生信研究\\Sepsis\\数据分析\\mROC\\E-MTAB-7581")

library(pROC) # 加载pROC包
library(ggplot2) # 调用ggplot2包以利用ggroc函数

data=read.table("SPSS.csv",sep=",",header=T,check.names=F,row.names = 1)

roc.list <- roc(Status ~ Age + SRS +APACHEII+ IRG_score, data = data)
ggroc1 <- ggroc(roc.list,
legacy.axes = TRUE);

ggroc1
ggroc2<-ggroc1 + xlab("1-specificity") + ylab("Sensitivity") +theme_bw()+ theme(panel.grid.major=element_line(colour=NA),

panel.grid.minor = element_blank()) + geom_segment(aes(x = 0,xend = 1,y = 0,yend = 1),col = "darkgrey",linetype = "dashed")

ggroc3<-ggroc2+

theme(legend.position=c(0.85,0.30))+#设置图例位置

theme(legend.text=element_text(size=10,face="plain"))+#设置图例字体格式

theme(legend.background=element_rect(fill="white",colour="black"))#图例填充白色，边框为黑色

ggroc3

ggroc4<-ggroc3+scale_y_continuous(expand=c(0,0))+scale_x_continuous(expand=c(0,0))

ggroc4

roc1 <- roc(data$Status,data$Age);roc1 # Build a ROC object and compute the AUC
roc2 <- roc(data$Status,data$SRS);roc2

roc3 <- roc(data$Status,data$APACHEII);roc3

roc4 <- roc(data$Status,data$HLA_score);roc4

ggroc4 + annotate("text", x=0.6, y=0.60, label="AUC = 0.570",size=3) +
    annotate("text", x=0.6, y=0.7, label="AUC = 0.675",size=3) +
    annotate("text", x=0.6, y=0.8, label="AUC = 0.694",size=3)+
    annotate("text", x=0.6, y=0.9, label="AUC = 0.694",size=3)

setwd("F:\\科研论文\\科研学术\\ICU生信研究\\Sepsis\\HLA\\数据分析\\mROC\\E-MTAB-7581")

library(pROC) # 加载pROC包
library(ggplot2) # 调用ggplot2包以利用ggroc函数

data=read.table("SPSS.txt",sep="\t",header=T,check.names=F,row.names = 1)

roc.list <- roc(Status ~ Age + MARS + HLA_score, data = data)
ggroc1 <- ggroc(roc.list,
legacy.axes = TRUE);

ggroc1
ggroc2<-ggroc1 + xlab("1-specificity") + ylab("Sensitivity") +theme_bw()+ theme(panel.grid.major=element_line(colour=NA),

panel.grid.minor = element_blank()) + geom_segment(aes(x = 0,xend = 1,y = 0,yend = 1),col = "darkgrey",linetype = "dashed")

ggroc3<-ggroc2+

theme(legend.position=c(0.85,0.20))+#设置图例位置

theme(legend.text=element_text(size=10,face="plain"))+#设置图例字体格式

theme(legend.background=element_rect(fill="white",colour="black"))#图例填充白色，边框为黑色

ggroc3

ggroc4<-ggroc3+scale_y_continuous(expand=c(0,0))+scale_x_continuous(expand=c(0,0))

ggroc4

roc1 <- roc(data$Status,data$Age);roc1 # Build a ROC object and compute the AUC
roc2 <- roc(data$Status,data$MARS);roc2

roc3 <- roc(data$Status,data$IRG_score);roc3

ggroc4+annotate("text",x=0.6,y=0.5,label="AUC=0.477",size=3)+

annotate("text",x=0.6,y=0.68,label="AUC=0.569",size=3)+

annotate("text",x=0.6,y=0.9,label="AUC=0.711",size=3)

**DCA**

setwd("F:\\科研论文\\科研学术\\ICU生信研究\\Sepsis\\HLA\\数据分析\\DCA")

source("dca.R")

library(nricens)

library(rms)

library(foreign)

dev=read.table("SPSS.txt",sep="\t",header=T,check.names=F,row.names = 1)

modelA <- glm(Status~B2M+HLA-DPA1+HLA-DQA1+TAP1+TAP2, data = dev, family = binomial(link="logit"),x=TRUE)

summary(modelA)

dev$IRG_score<- predict(newdata=dev,modelA,"response")

modelB <- glm(Status ~Age, data = dev, family = binomial(link="logit"),x=TRUE)

summary(modelB)

dev$Age<- predict(newdata=dev,modelB,"response")

modelC <- glm(Status ~SRS, data = dev, family = binomial(link="logit"),x=TRUE)

summary(modelC)

dev$SRS<- predict(newdata=dev,modelC,"response")

#Decision Curve Analysis

dca(data=dev, outcome="Status", predictors=c("IRG_score", "Age","SRS"),smooth="TRUE", probability=c("TRUE", "TRUE","TRUE"))

setwd("F:\\科研论文\\科研学术\\ICU生信研究\\Sepsis\\HLA\\数据分析\\DCA")

source("dca.R")

library(nricens)

library(rms)

library(foreign)

dev=read.table("SPSS.csv",sep=",",header=T,check.names=F,row.names = 1)

modelA <- glm(Status~B2M+HLA-DPA1+HLA-DQA1+TAP1+TAP2, data = dev, family = binomial(link="logit"),x=TRUE)

summary(modelA)

dev$IRG_score<- predict(newdata=dev,modelA,"response")

modelB <- glm(Status ~Age, data = dev, family = binomial(link="logit"),x=TRUE)

summary(modelB)

dev$Age<- predict(newdata=dev,modelB,"response")

modelC <- glm(Status ~SRS, data = dev, family = binomial(link="logit"),x=TRUE)

summary(modelC)

dev$SRS<- predict(newdata=dev,modelC,"response")

modelD <- glm(Status ~APACHEII, data = dev, family = binomial(link="logit"),x=TRUE)

summary(modelD)

dev$APACHEII<- predict(newdata=dev,modelD,"response")

#Decision Curve Analysis

dca(data=dev, outcome="Status", predictors=c("IRG_score","Age","SRS","APACHEII"),smooth="TRUE", probability=c("TRUE", "TRUE","TRUE","TRUE"))

**ssGSEA**

**Immune Cell**

library(GSVA)

library(limma)

library(GSEABase)

setwd("F:\\科研论文\\科研学术\\ICU生信研究\\Sepsis\\HLA\\数据分析\\ICI\\E-MTAB-4421") #设置工作目录

inputFile="geneMatrix.txt" #输入文件

gmtFile="ImmuneCell.gmt" #GMT文件

#读取输入文件，并对输入文件处理

rt=read.table(inputFile,sep="\t",header=T,check.names=F)

rt=as.matrix(rt)

rownames(rt)=rt[,1]

exp=rt[,2:ncol(rt)]

dimnames=list(rownames(exp),colnames(exp))

mat=matrix(as.numeric(as.matrix(exp)),nrow=nrow(exp),dimnames=dimnames)

mat=avereps(mat)

mat=mat[rowMeans(mat)>0,]

geneSet=getGmt(gmtFile,

geneIdType=SymbolIdentifier())

#ssgsea分析

ssgseaScore=gsva(mat, geneSet, method='ssgsea', kcdf='Gaussian', abs.ranking=TRUE)

#定义ssGSEA score矫正函数

normalize=function(x){

return((x-min(x))/(max(x)-min(x)))}

#对ssGSEA score进行矫正

ssgseaOut=normalize(ssgseaScore)

ssgseaOut=rbind(id=colnames(ssgseaOut),ssgseaOut)

write.table(ssgseaOut,file="ssGSEA.txt",sep="\t",quote=F,col.names=F)

**CIBERSORTx**

setwd("F:\\科研论文\\科研学术\\ICU生信研究\\Sepsis\\HLA\\数据分析\\ICI\\E-MTAB-4421") #设置工作目录

library(vioplot) #引用包

library(limma)

rt=read.table("CIBERSORTx.txt",sep="\t",header=T,row.names=1,check.names=F) #读取输入文件

normal=140 #正常样品数目

tumor=125 #肿瘤样品数目

pdf("CIBERSORT.pdf",height=8,width=15) #保存图片的文件名称

par(las=1,mar=c(10,6,3,3))

x=c(1:ncol(rt))

y=c(1:ncol(rt))

plot(x,y,

xlim=c(0,63),ylim=c(min(rt),max(rt)+0.02),

main="",xlab="", ylab="Fraction",

pch=21,

col="white",

xaxt="n")

#对每个免疫细胞循环，绘制vioplot，正常用绿色表示，肿瘤用红色表示

for(i in 1:ncol(rt)){

normalData=rt[1:normal,i]

tumorData=rt[(normal+1):(normal+tumor),i]

vioplot(normalData,at=3*(i-1),lty=1,add = T,col = 'green')

vioplot(tumorData,at=3*(i-1)+1,lty=1,add = T,col = 'red')

wilcoxTest=wilcox.test(normalData,tumorData)

p=round(wilcoxTest$p.value,3)

mx=max(c(normalData,tumorData))

lines(c(x=3*(i-1)+0.2,x=3*(i-1)+0.8),c(mx,mx))

text(x=3*(i-1)+0.5,y=mx+0.02,labels=ifelse(p<0.001,paste0("p<0.001"),paste0("p=",p)),cex = 0.8)

text(seq(1,64,3),-0.05,xpd = NA,labels=colnames(rt),cex = 1,srt = 45,pos=2)

}

dev.off()

**Immune Cell**

setwd("F:\\科研论文\\科研学术\\ICU生信研究\\Sepsis\\HLA\\数据分析\\ICI\\E-MTAB-4421") #设置工作目录

library(vioplot) #引用包

library(limma)

rt=read.table("ssGSEA.txt",sep="\t",header=T,row.names=1,check.names=F) #读取输入文件

normal=140 #正常样品数目

tumor=125 #肿瘤样品数目

pdf("ssGSEA.pdf",height=8,width=18) #保存图片的文件名称

par(las=1,mar=c(10,6,3,3))

x=c(1:ncol(rt))

y=c(1:ncol(rt))

plot(x,y,

xlim=c(0,76),ylim=c(min(rt),max(rt)+0.02),

main="",xlab="", ylab="Immune infiltration",

pch=21,

col="white",

xaxt="n")

#对每个免疫细胞循环，绘制vioplot，正常用绿色表示，肿瘤用红色表示

for(i in 1:ncol(rt)){

normalData=rt[1:normal,i]

tumorData=rt[(normal+1):(normal+tumor),i]

vioplot(normalData,at=3*(i-1),lty=1,add = T,col = 'green')

vioplot(tumorData,at=3*(i-1)+1,lty=1,add = T,col = 'red')

wilcoxTest=wilcox.test(normalData,tumorData)

p=round(wilcoxTest$p.value,3)

mx=max(c(normalData,tumorData))

lines(c(x=3*(i-1)+0.2,x=3*(i-1)+0.8),c(mx,mx))

text(x=3*(i-1)+0.5,y=mx+0.02,labels=ifelse(p<0.001,paste0("p<0.001"),paste0("p=",p)),cex = 0.8)

text(seq(1,76,3),-0.1,xpd = NA,labels=colnames(rt),cex = 1,srt = 45,pos=2)

}

dev.off()

**Barplot**

setwd("F:\\科研论文\\科研学术\\ICU生信研究\\Sepsis\\数据分析\\ICI\\GSE65682")

input="CIBERSORT.txt"

outpdf="Barplot.pdf"

data <- read.table(input,header=T,sep="\t",check.names=F,row.names=1)

data=t(data)

col=rainbow(nrow(data),s=0.7,v=0.7)

pdf(outpdf,height=10,width=22)

par(las=1,mar=c(8,5,4,16),mgp=c(3,0.1,0),cex.axis=1.5)

a1 = barplot(data,col=col,yaxt="n",ylab="Relative Percent",xaxt="n",cex.lab=1.8)

a2=axis(2,tick=F,labels=F)

axis(2,a2,paste0(a2*100,"%"))

axis(1,a1,labels=F)

par(srt=60,xpd=T);text(a1,-0.02,colnames(data),adj=1,cex=0.6);par(srt=0)

ytick2 = cumsum(data[,ncol(data)])

ytick1 = c(0,ytick2[-length(ytick2)])

legend(par('usr')[2]*0.98,par('usr')[4],legend=rownames(data),col=col,pch=15,bty="n",cex=1.3)

dev.off()

**Signaling Pathway**

library(GSVA)

library(limma)

library(GSEABase)

setwd("F:\\科研论文\\科研学术\\ICU生信研究\\Sepsis\\数据分析\\ImmuneSP\\E-MTAB-4421") #设置工作目录

inputFile="geneMatrix.txt" #输入文件

gmtFile="ImmuneSP.gmt" #GMT文件

#读取输入文件，并对输入文件处理

rt=read.table(inputFile,sep="\t",header=T,check.names=F)

rt=as.matrix(rt)

rownames(rt)=rt[,1]

exp=rt[,2:ncol(rt)]

dimnames=list(rownames(exp),colnames(exp))

mat=matrix(as.numeric(as.matrix(exp)),nrow=nrow(exp),dimnames=dimnames)

mat=avereps(mat)

mat=mat[rowMeans(mat)>0,]

geneSet=getGmt(gmtFile,

geneIdType=SymbolIdentifier())

#ssgsea分析

ssgseaScore=gsva(mat, geneSet, method='ssgsea', kcdf='Gaussian', abs.ranking=TRUE)

#定义ssGSEA score矫正函数

normalize=function(x){

return((x-min(x))/(max(x)-min(x)))}

#对ssGSEA score进行矫正

ssgseaOut=normalize(ssgseaScore)

ssgseaOut=rbind(id=colnames(ssgseaOut),ssgseaOut)

write.table(ssgseaOut,file="ssGSEAS.txt",sep="\t",quote=F,col.names=F)

**ImP**

setwd("F:\\科研论文\\科研学术\\ICU生信研究\\Sepsis\\HLA\\数据分析\\ImmuneSP\\E-MTAB-4421") #设置工作目录

library(vioplot) #引用包

library(limma)

rt=read.table("ssGSEAS.txt",sep="\t",header=T,row.names=1,check.names=F) #读取输入文件

normal=140 #正常样品数目

tumor=125 #肿瘤样品数目

pdf("ssGSEAS.pdf",height=8,width=12) #保存图片的文件名称

par(las=1,mar=c(10,6,3,3))

x=c(1:ncol(rt))

y=c(1:ncol(rt))

plot(x,y,

xlim=c(0,37),ylim=c(min(rt),max(rt)+0.02),

main="",xlab="", ylab="Score",

pch=21,

col="white",

xaxt="n")

#对每个免疫细胞循环，绘制vioplot，正常用绿色表示，肿瘤用红色表示

for(i in 1:ncol(rt)){

normalData=rt[1:normal,i]

tumorData=rt[(normal+1):(normal+tumor),i]

vioplot(normalData,at=3*(i-1),lty=1,add = T,col = 'blue')

vioplot(tumorData,at=3*(i-1)+1,lty=1,add = T,col = 'red')

wilcoxTest=wilcox.test(normalData,tumorData)

p=round(wilcoxTest$p.value,3)

mx=max(c(normalData,tumorData))

lines(c(x=3*(i-1)+0.2,x=3*(i-1)+0.8),c(mx,mx))

text(x=3*(i-1)+0.5,y=mx+0.02,labels=ifelse(p<0.001,paste0("p<0.001"),paste0("p=",p)),cex = 0.8)

text(seq(1,37,3),-0.1,xpd = NA,labels=colnames(rt),cex = 1,srt = 45,pos=2)

}

dev.off()

**Cytokine**

setwd("F:\\科研论文\\科研学术\\ICU生信研究\\Sepsis\\HLA\\数据分析\\Cytokine\\E-MTAB-4421")

#设置工作目录

library(vioplot) #引用包

library(limma)

rt=read.table("CKExp.txt",sep="\t",header=T,row.names=1,check.names=F) #读取输入文件

normal=140 #正常样品数目

tumor=125 #肿瘤样品数目

pdf("CK.pdf",height=8,width=12) #保存图片的文件名称

par(las=1,mar=c(10,6,3,3))

x=c(1:ncol(rt))

y=c(1:ncol(rt))

plot(x,y,

xlim=c(0,47),ylim=c(min(rt),max(rt)+0.02),

main="",xlab="", ylab="Expression",

pch=21,

col="white",

xaxt="n")

#对每个免疫细胞循环，绘制vioplot，正常用绿色表示，肿瘤用红色表示

for(i in 1:ncol(rt)){

normalData=rt[1:normal,i]

tumorData=rt[(normal+1):(normal+tumor),i]

vioplot(normalData,at=3*(i-1),lty=1,add = T,col = 'white')

vioplot(tumorData,at=3*(i-1)+1,lty=1,add = T,col = 'red')

wilcoxTest=wilcox.test(normalData,tumorData)

p=round(wilcoxTest$p.value,3)

mx=max(c(normalData,tumorData))

lines(c(x=3*(i-1)+0.2,x=3*(i-1)+0.8),c(mx,mx))

text(x=3*(i-1)+0.5,y=mx+0.2,labels=ifelse(p<0.001,paste0("p<0.001"),paste0("p=",p)),cex = 0.8)

text(seq(1,47,3),0.8,xpd = NA,labels=colnames(rt),cex = 1,srt = 45,pos=2)

}

dev.off()

setwd("F:\\科研论文\\科研学术\\ICU生信研究\\Sepsis\\HLA\\数据分析\\Cytokine\\GSE63042")

#设置工作目录

library(vioplot) #引用包

library(limma)

rt=read.table("CKExp.txt",sep="\t",header=T,row.names=1,check.names=F)

rt=log2(rt[,1:ncol(rt)]+1)

#读取输入文件

normal=58 #正常样品数目

tumor=48 #肿瘤样品数目

pdf("CK.pdf",height=7,width=10) #保存图片的文件名称

par(las=1,mar=c(10,6,3,3))

x=c(1:ncol(rt))

y=c(1:ncol(rt))

plot(x,y,

xlim=c(0,29),ylim=c(min(rt),max(rt)+0.02),

main="",xlab="", ylab="Expression",

pch=21,

col="white",

xaxt="n")

#对每个免疫细胞循环，绘制vioplot，正常用绿色表示，肿瘤用红色表示

for(i in 1:ncol(rt)){

normalData=rt[1:normal,i]

tumorData=rt[(normal+1):(normal+tumor),i]

vioplot(normalData,at=3*(i-1),lty=1,add = T,col = 'white')

vioplot(tumorData,at=3*(i-1)+1,lty=1,add = T,col = 'red')

wilcoxTest=wilcox.test(normalData,tumorData)

p=round(wilcoxTest$p.value,3)

mx=max(c(normalData,tumorData))

lines(c(x=3*(i-1)+0.2,x=3*(i-1)+0.8),c(mx,mx))

text(x=3*(i-1)+0.5,y=mx+0.2,labels=ifelse(p<0.001,paste0("p<0.001"),paste0("p=",p)),cex = 0.8)

text(seq(1,29,3),-1,xpd = NA,labels=colnames(rt),cex = 1,srt = 45,pos=2)

}

dev.off()

**GSVA**

library(GSEABase)

library(GSVA)

expFile="geneMatrix.txt" #表达输入文件

gmtFile="KEGG.gmt" #基因集文件

setwd("F:\\科研论文\\科研学术\\ICU生信研究\\Sepsis\\数据分析\\GSVA\\E-MTAB-4421") #设置工作目录

rt=read.table(expFile, header=T, sep="\t", check.names=F)

rt=as.matrix(rt)

rownames(rt)=rt[,1]

exp=rt[,2:ncol(rt)]

dimnames=list(rownames(exp), colnames(exp))

data=matrix(as.numeric(as.matrix(exp)), nrow=nrow(exp), dimnames=dimnames)

data=avereps(data)

#GSVA分析

geneSets=getGmt(gmtFile, geneIdType=SymbolIdentifier())

gsvaResult=gsva(data,

geneSets,

min.sz=10,

max.sz=500,

verbose=TRUE,

parallel.sz=1)

gsvaOut=rbind(id=colnames(gsvaResult), gsvaResult)

write.table(gsvaOut, file="GSVA.txt", sep="\t", quote=F, col.names=F)

**差异分析**

library(limma)

setwd("F:\\科研论文\\科研学术\\ICU生信研究\\Sepsis\\HLA\\数据分析\\GSVA\\E-MTAB-4421") #设置工作目录

rt=read.table("GSVA.txt", header=T, sep="\t", row.names=1)

logFCcutoff=0.3

adjPvalueCutoff=0.05

type=c( rep("con",140),rep("treat",125) )

design=model.matrix(~ type)

colnames(design)=c("con", "treat")

fit=lmFit(rt, design)

fit=eBayes(fit)

dif=topTable(fit, coef="con", number=Inf,adjust.method="holm")

dif[1:10,1:5]

write.table(dif,file="Diff.txt",sep="\t",quote=F)

**DotChart**

setwd("F:\\科研论文\\科研学术\\ICU生信研究\\Sepsis\\HLA\\数据分析\\GSVA\\E-MTAB-4421")

mydata=read.table("SP.txt", header=T, sep="\t")

library(ggpubr)

ggdotchart(mydata, x = "id", y = "logFC",

color = "Group",

palette = c("#FC4E07", "#00AFBB"),

sorting = "descending",

add = "segments",

add.params = list(color = "lightgray", size = 1),

group = "Group",

dot.size = 6,

font.label = list(color = "white", size = 8,

vjust = 0.5),

xlab="",

ylab="logFC" ,

ggtheme = theme_pubr())+

geom_hline(yintercept = 0, linetype = 2, color = "lightgray")

**Cor Cell**

library(limma)

library(reshape2)

library(ggplot2)

immFile="ssGSEA.txt" #免疫细胞浸润结果文件

riskFile="RiskScore.txt" #风险文件

setwd("F:\\科研论文\\科研学术\\ICU生信研究\\Sepsis\\HLA\\数据分析\\Cor Cell\\E-MTAB-4421")

#设置工作目录

#读取免疫细胞结果文件，并对数据进行整理

data=read.table(immFile, header=T, sep="\t", check.names=F, row.names=1)

risk=read.table(riskFile, header=T, sep="\t", check.names=F, row.names=1)

sameSample=intersect(row.names(data), row.names(risk))

data=data[sameSample,,drop=F]

risk=risk[sameSample,,drop=F]

#相关性分析

outTab=data.frame()

for(immune in colnames(data)){

for(gene in colnames(risk)){

x=as.numeric(data[,immune])

y=as.numeric(risk[,gene])

corT=cor.test(x,y,method="spearman")

cor=corT$estimate

pvalue=corT$p.value

text=ifelse(pvalue<0.001,"***",ifelse(pvalue<0.01,"**",ifelse(pvalue<0.05,"*","")))

outTab=rbind(outTab,cbind(Gene=gene, Immune=immune, cor=cor, text, pvalue))

}

}

write.table(outTab,file="Cor.txt",sep="\t",row.names=F,quote=F)

#绘制相关性热图

outTab$cor=as.numeric(outTab$cor)

pdf(file="Cor.pdf", width=6, height=8)

ggplot(outTab, aes(Immune, Gene)) +

geom_tile(aes(fill = cor), colour = "grey", size = 1)+

scale_fill_gradient2(low = "#5C5DAF", mid = "white", high = "#EA2E2D") +

geom_text(aes(label=text),col ="black",size = 3) +

theme_minimal() + #去掉背景

theme(axis.title.x=element_blank(), axis.ticks.x=element_blank(), axis.title.y=element_blank(),

axis.text.x = element_text(angle = 60, hjust = 1, size = 10, face = "bold"), #x轴字体

axis.text.y = element_text(size = 10, face = "bold")) + #y轴字体

labs(fill =paste0("*** p<0.001","\n", "** p<0.01","\n", " * p<0.05","\n", "\n","Correlation")) + #设置图例

scale_x_discrete(position = "bottom") #X轴名称显示位置

dev.off()

**Cor SP**

library(limma)

library(reshape2)

library(ggplot2)

immFile="ssGSEA.txt" #免疫细胞浸润结果文件

riskFile="RiskScore.txt" #风险文件

setwd("F:\\科研论文\\科研学术\\ICU生信研究\\Sepsis\\HLA\\数据分析\\Cor SP\\E-MTAB-4421")

#设置工作目录

#读取免疫细胞结果文件，并对数据进行整理

data=read.table(immFile, header=T, sep="\t", check.names=F, row.names=1)

risk=read.table(riskFile, header=T, sep="\t", check.names=F, row.names=1)

sameSample=intersect(row.names(data), row.names(risk))

data=data[sameSample,,drop=F]

risk=risk[sameSample,,drop=F]

#相关性分析

outTab=data.frame()

for(immune in colnames(data)){

for(gene in colnames(risk)){

x=as.numeric(data[,immune])

y=as.numeric(risk[,gene])

corT=cor.test(x,y,method="spearman")

cor=corT$estimate

pvalue=corT$p.value

text=ifelse(pvalue<0.001,"***",ifelse(pvalue<0.01,"**",ifelse(pvalue<0.05,"*","")))

outTab=rbind(outTab,cbind(Gene=gene, Immune=immune, cor=cor, text, pvalue))

}

}

write.table(outTab,file="Cor.txt",sep="\t",row.names=F,quote=F)

#绘制相关性热图

outTab$cor=as.numeric(outTab$cor)

pdf(file="Cor.pdf", width=6, height=4)

ggplot(outTab, aes(Gene, Immune)) +

geom_tile(aes(fill = cor), colour = "grey", size = 1)+

scale_fill_gradient2(low = "#5C5DAF", mid = "white", high = "#EA2E2D") +

geom_text(aes(label=text),col ="black",size = 3) +

theme_minimal() + #去掉背景

theme(axis.title.x=element_blank(), axis.ticks.x=element_blank(), axis.title.y=element_blank(),

axis.text.x = element_text(angle = 60, hjust = 1, size = 10, face = "bold"), #x轴字体

axis.text.y = element_text(size = 10, face = "bold")) + #y轴字体

labs(fill =paste0("*** p<0.001","\n", "** p<0.01","\n", " * p<0.05","\n", "\n","Correlation")) + #设置图例

scale_x_discrete(position = "bottom") #X轴名称显示位置

dev.off()

**Cor Gene**

library(reshape2)

library(ggpubr)

library(ggExtra)

library(pheatmap)

setwd("F:\\科研论文\\科研学术\\ICU生信研究\\Sepsis\\HLA\\数据分析\\Cor Gene\\GSE65682")

rt=read.table("Cor.txt", header=T, sep="\t", check.names=F, row.names=1)

#读取输入文件

#绘制基因与免疫细胞相关性的散点图

x=as.numeric(rt[,"HLA score"])

y=as.numeric(rt[,"IL10/TNF"])

df1=as.data.frame(cbind(x,y))

p1=ggplot(df1, aes(x, y)) +

xlab("HLA score") +

ylab("IL10/TNF") +

geom_point() + geom_smooth(method="lm",formula = y ~ x) + theme_bw()+

stat_cor(method = 'spearman', aes(x =x, y =y))

p2=ggMarginal(p1, type="density", xparams=list(fill = "orange"), yparams=list(fill = "blue"))

p2

pdf(file=paste0("IL10.pdf"),width=6,height=6)

print(p2)

dev.off()

**GSE11375**

setwd("F:\\科研论文\\科研学术\\ICU生信研究\\Sepsis\\HLA\\Frontiers in Immunology\\GSE11375")

inputfile1="intersectGene.txt" #生存时间数据

inputfile2="geneMatrix.txt" #差异基因表达数据

time_data<-read.table(inputfile1,header = T,sep = "\t",check.names = F)

geneEXP<-read.table(inputfile2,header = T,sep = "\t",check.names = F)

head(time_data)

head(geneEXP)

merger_data<-merge(time_data,geneEXP,by="id")

write.table(merger_data,"HLAExp.txt",sep = "\t",row.names = F,quote = F)

setwd("F:\\科研论文\\科研学术\\ICU生信研究\\Sepsis\\HLA\\Frontiers in Immunology\\GSE11375")

lncRNA<-read.table("HLAExp.txt",header=T,sep="\t",row.names = 1,check.names = F,stringsAsFactors = F)

lncRNAEXP=log2(lncRNA[,1:ncol(lncRNA)]+1)

lncRNA=cbind(lncRNAEXP)

write.table(cbind(id=rownames(cbind(lncRNA[,1:5])),cbind(lncRNA[,1:5])),"HLA.txt",sep="\t",quote=F,row.names=F)

**dROC**

library(rms)

library(foreign)

setwd("F:\\科研论文\\科研学术\\ICU生信研究\\Sepsis\\HLA\\Frontiers in Immunology\\GSE11375")

data=read.table("dROC.txt",sep="\t",header=T,check.names=F,row.names = 1)

ddist <- datadist(data)

options(datadist='ddist')

modelA <- glm(Status~., data = data, family = binomial(link="logit"))

summary(modelA)

cbind(coef= coef(modelA),confint(modelA))

exp(cbind(OR= coef(modelA),confint(modelA)))

Score<- predict(newdata=data,modelA,"response")

write.table(cbind(id=rownames(cbind(data[,1:6],Score)),cbind(data[,1:6],Score)),"RiskScore.txt",sep="\t",quote=F,row.names=F)

data=read.table("RiskScore.txt",sep="\t",header=T,check.names=F,row.names = 1)

library(pROC)

gmodelA <- roc(Status~Score, data = data,smooth=F)

plot(gmodelA, print.auc=TRUE, print.thres=TRUE,main = "ROC CURVE", col= "blue",print.thres.col="blue",identity.col="blue",

identity.lty=1,identity.lwd=1)

setwd("F:\\科研论文\\科研学术\\ICU生信研究\\Sepsis\\HLA\\Frontiers in Immunology\\GSE11375")

data=read.table("RiskScore.txt",sep="\t",header=T,check.names=F,row.names = 1)

library(pROC)

gmodelA <- roc(Status~Score, data = data,smooth=F)

pdf(file="dROC.pdf", width=7, height=6)

plot(gmodelA, print.auc=TRUE, main = "ROC CURVE", col= "blue",print.thres.col="blue",identity.col="blue",

identity.lty=1,identity.lwd=1)

dev.off()
