# Supplementary material for: Construction of an HLA Classifier for Early Diagnosis, Prognosis, and Recognition of Immunosuppression in Sepsis by Multiple Transcriptome Datasets
Source: Front Physiol. 2022 May 24;13:870657. doi: 10.3389/fphys.2022.870657 (PMC9171028; doi:10.3389/fphys.2022.870657)
Supplement: Supplementary file 17 [file DataSheet1.docx]

Supplementary Material 4

B2M

Forward primer AGCAGCATCATGGAGGTTTG

Reverse primer AGCCCTCCTAGAGCTACCTG

HLA-DQA1

Forward primer TCCTTGTGTTCCCACCCTTG

Reverse primer CAAGGGCAGACGGTATCCAT

HLA-DPA1

Forward primer CCGTCCCTGGAAAAGTGCTA

Reverse primer CTCCCCTGTTGGTCTATGCG

TAP1

Forward primer

Reverse primer CAGAGCACAGTCTCCGTTGT

TAP2

Forward primer TACGTGGCCACTAGGTGGTA

Reverse primer TTCAAAAGCTCAGCCCCTGG
